# NLRX1 Drives Prostate Cancer Progression Through Activation of AKT and ERK Signaling Pathways

**DOI:** 10.7150/ijbs.126054

**Published:** 2026-04-16

**Authors:** Varsha Rathore, Ching-Yuan Cheng, Duen-Yi Huang, Shao-Peng Chen, Liang Huan Wu, Jitendra Maharana, Chuang-Rung Chang, Wan-Wan Lin

**Affiliations:** 1 Chemical Biology and Molecular Biophysics, Taiwan International Graduate Program, Academia Sinica, Taipei 115201, Taiwan.; 2 Institute of Biotechnology, College of Life Sciences and Medicine, National Tsing Hua University, Hsinchu 300044, Taiwan.; 3 Department of Pharmacology, College of Medicine, National Taiwan University, Taipei 10051, Taiwan.; 4 Department of Ophthalmology, Cardinal Tien Hospital, New Taipei City 23148, Taiwan.; 5 Institute of Bioinformatics and Structural Biology, College of Life Sciences and Medicine, National Tsing Hua University, Hsinchu 300044, Taiwan.; 6 Graduate Institute of Medical Sciences, Taipei Medical University, Taipei 110, Taiwan.

**Keywords:** Prostate cancer, NLRX1, AKT, Apoptosis, Invasion, Migration

## Abstract

NLRX1, a mitochondrial NOD-like receptor (NLR) family protein, is a non-inflammasome-forming protein with diverse roles in cancer. While NLRX1 has been recognized as a tumor suppressor in colorectal and hepatocellular carcinomas, it appears to act as a tumor promoter in breast and head and neck cancers. This study explored the role of NLRX1 in prostate cancer (PCa), examining its impact on cell proliferation, apoptosis, migration, invasion, and tumor progression, as well as associated molecular mechanisms. Using TCGA data, the association between NLRX1 expression and PCa prognosis was evaluated. NLRX1 expression was upregulated under serum-free stress conditions. Silencing NLRX1 reduced cell proliferation in PC3 cells, but not in LNCaP cells. Additionally, NLRX1 knockdown inhibited migration and invasion, while promoting apoptosis under serum-free conditions. Mechanistically, NLRX1 knockdown reduced AKT and ERK phosphorylation in response to serum deprivation, EGF, and TGF-β, without affecting PDK1 activity under serum deprivation. Pharmacological data showed AKT and ERK as key regulators of viability and invasion, with AKT critical for growth and migration. Co-immunoprecipitation, confocal microscopic examination, domain binding, structural modeling, and molecular dynamics revealed a stable interaction between NLRX1's LRR domain and AKT's PH domain. NLRX1 facilitated cell proliferation, migration, invasion, and resistance to serum-free stress through direct interaction with AKT, highlighting NLRX1 as a promising biomarker for PCa progression.

## Introduction

NOD-like receptor X1 (NLRX1), often called NOD5, NOD9, and CLR11.3, is a member of the NOD-like receptor family. Like other NLRs, NLRX1 contains a central NACHT domain that mediates nucleotide binding and oligomerization, supporting its role as a scaffolding protein, as well as a C-terminal LRR domain. In addition, the first 39 amino acids at the N-terminus of NLRX1 comprise a unique and unconventional mitochondrial targeting sequence 1. Of all the NOD-like receptors, NLRX1 is the only one that targets the mitochondria 2 and is located in the outer mitochondrial membrane 1, inner mitochondrial membrane 3, and the mitochondrial matrix 1,4,5. NLRX1 is a highly conserved receptor across species and is widely detected in all tissues, with the highest expression levels in the heart, muscle, and mammary gland 6. Although NLRX1 is a receptor, its ligands are still unidentified.

Most research has focused on the crucial roles of NLRX1 in immune cells and the host-pathogen interaction 7-9. NLRX1 inhibits type I interferon production mediated by mitochondrial antiviral signaling protein (MAVS) 10-12. This action confers NLRX1 to be a brake on mitochondria-mediated antiviral immunity 12. Nevertheless, a recent study shows that NLRX1 may sequester STING during viral infection and functions upstream to mediate the cGAS-STING pathway in the brain 13. In addition, NLRX1 was shown to serve as a suppressor of the NF-κB pathway by targeting TRAF6 and interrupting TRAF6-IKK interaction 10. Nevertheless, NLRX1 can also amplify the NF-κB pathway by producing intracellular reactive oxygen species (ROS) 14. NLRX1 deficiency causes more severe pathological phenomena by boosting activation of NF-κB downstream signaling, and in turn, exacerbating neural injury 15, osteoarthritis 16, inflammatory bowel disease 17, ischemia-reperfusion injury 18, CNS inflammation 19, and spontaneous autoimmunity 20. Nevertheless, NLRX1 loss in hepatocytes stimulates fatty acid oxidation, preventing hepatic steatosis and metabolic syndrome 21.

NLRX1's function in various malignancies has been the subject of recent research, which suggests that it may either promote or suppress tumor growth 22, while the detailed underlying mechanisms remain elusive. In some cancer cells, NLRX1 is downregulated and is suggested to be a tumor suppressor. These include colorectal cancer 23-25, hepatocellular carcinoma 26, gastric cancer 25,27, histiocytic sarcoma 28, esophageal squamous cell carcinoma 29, and pancreatic cancer 30*.* Furthermore, NLRX1 downregulation enhances colon cancer cell capacity to proliferate, form tumors, and invade. Moreover, elevated NLRX1 adversely affects basal respiration and mitochondrial ATP-linked respiration in colon cancer cells 23. In hepatocellular carcinoma, NLRX1 can inhibit epithelial-mesenchymal transition (EMT) by decreasing AKT phosphorylation 31. NLRX1 also sensitizes cells to TNF-α-induced caspase-8-dependent cell apoptosis by associating with TNF receptor complex II, enhancing the active subunit of caspase-8 localizing to the mitochondria and mitochondrial ROS (mtROS) generation, as well as decreasing mitochondrial complex I and complex III activities upon TNF-α stimulation in cancer cells 32.

Despite most studies highlighting NLRX1 as a tumor suppressor, some studies show opposite findings. Clinical breast cancer analyses indicate that NLRX1 is elevated in basal-like and metastatic carcinomas compared with non-basal-like and primary breast tumors 33. In triple-negative breast cancer cells, NLRX1 augments cell proliferation, migration, and metastasis but attenuates ROS-mediated cell death and mitochondrial respiratory capacity 30. Loss of NLRX1 decreases oxidative phosphorylation-dependent cell proliferation and migration of triple-negative breast cancer cells in the presence of TNF-α 33. The controversial roles of NLRX1 suggest context-dependent, cancer cell-specific effects. However, in addition to cancer type specificity, the tumor microenvironment regulated by NLRX1 also represents a critical factor contributing to its inconsistent roles in cancer progression 30,33.

To date, the role of NLRX1 in prostate cancer (PCa) remains unclear. In this study, we employed NLRX1 knockdown in PC3 and LNCaP cell lines to investigate its function in PCa cells. Our findings indicate that NLRX1 is associated with PCa prognosis and contributes to cell proliferation, migration, invasion, and resistance to serum deprivation-induced cell death. The *in vivo* xenograft study further demonstrates that NLRX1 promotes tumor growth in PC3 cells. Mechanistically, NLRX1 exerts its tumorigenic effects in PCa cells by positively regulating AKT activation through direct protein-protein interaction.

## Materials and Methods

### Reagents and antibodies

RPMI medium (Cat. No. 23400-021) and trypsin-EDTA were acquired from Gibco (Carlsbad, CA, USA). Fetal bovine serum was provided by RMBIO (Gregs Way, MT, USA). Phosphate-buffered saline (PBS) and mitomycin C were sourced from Sigma-Aldrich (St. Louis, MO, USA). Penicillin-streptomycin-amphotericin B was acquired from Biological Industries (Kibbutz Beit Haemek, Israel). AKT inhibitor VIII and ERK inhibitor U0126 were procured from MedChemExpress (Monmouth Junction, NJ, USA). zVAD-FMK, N-acetyl cysteine (NAC), necrostatin-1 (Nec-1), dichlorodihydrofluorescein diacetate (H_2_DCFDA), and MitoSOX were obtained from Sigma-Aldrich Co (St Louis, MO, USA). MitoTracker was purchased from Thermo Fisher Scientific (Waltham, MA, USA). Puromycin was supplied by Thermo Fisher Scientific (Waltham, MA, USA). Antibodies against NLRX1 (sc-374514), ERK (sc-154), cyclin B (sc-1662), cyclin D1 (sc-246), PDK1 (sc-3062), PLK4 (sc-100413), and β-actin (sc-47778) were obtained from Santa Cruz Biotechnology (Dallas, TX, USA). MitoTracker-Red CMXRos (#9082P), Alexa Fluor® 647 Conjugate AKT (#5186), and antibodies against p-AKT (Ser473) (#4060S), AKT (#9272S), p-ERK (#9101), p-PDK1 (Ser241) (#3061S), p-mTOR (Ser2481) (#2974), and mTOR (#2972S) were purchased from Cell Signaling Technology (Beverly, MA, USA).

### Cell culture

Human PCa cell lines PC3 and LNCaP were maintained in RPMI medium enriched with 10% fetal bovine serum (FBS) and 1% penicillin-streptomycin-amphotericin B. Cultures were grown at 37 °C in a humidified incubator containing 5% CO₂. Serum starvation was achieved by culturing the cells in the same medium without FBS.

### Establishment of stable NLRX1 knockdown cell lines

To generate stable cell lines, lentiviral supernatants prepared in Opti-MEM medium supplemented with 8 µg/ml polybrene were used to transfect cells at 70% confluence. Lentiviral shRNA TRCN0130325 (GAGTCTGGAATCTCCAAGTTA) for silencing NLRX1 and shl.uc.976 RNA TRCN0072249 (GCGGTTGCCAAGAGGTTCCAT) for the control group was received from RNAiCore Academia Sinica. Following a 24 h transduction, the cells were subjected to a week of puromycin (2.5 µg/ml) selection. Subsequently, they were maintained in puromycin (1 µg/ml)-containing RPMI medium to sustain the stable PC3 and LNCaP cell lines with NLRX1 silencing.

### Analysis of PCa genomics data

The Gene Expression Omnibus (GEO) website was used to collect NLRX1 expression data from six GEO datasets: GSE21034, GSE29079, GSE46602, GSE119195, GSE104749, and GSE54460. Additionally, gene expression data for PCa were sourced from the Cancer Genome Atlas (TCGA) dataset. The UCSC Xena browser was employed to analyze RNA-sequencing data from the Genotype-Tissue Expression Project (GTEx) for healthy prostate tissue and from the TCGA-Prostate Adenocarcinoma (PRAD) for tumor tissue. To understand the NLRX1 gene mutation in PCa, the cBioPortal (accessed March 3, 2024) was utilized. We explored the copy number alteration (CNA), such as mutation, deep deletion, and amplification, in ten PCa datasets. Furthermore, the mutation landscape and mutation site of NLRX1 in PCa were also explored.

### Correlation between NLRX1 expression and PCa patients' survival

We used the TCGA-PRAD in the UCSC Xena 34 browser dataset to correlate the NLRX1 expression with overall survival. We used the GEO dataset GSE54460 from the Prostate Cancer Database (PCaDB) tool (http://bioinfo.jialab-ucr.org/PCaDB/, assessed on Feb 9th, 2026) to correlate the NLRX1 expression with relapse-specific survival. Then, we executed Kaplan-Meier plotting using a KM plotter with auto-select best cutoff 35. We also used the cBioPortal website to evaluate the association of the NLRX1 mutation with patients' prognosis.

### Tumor immune infiltration and immune checkpoints analysis

TIMER 2.0 (Tumor Immune Estimation Resource 2.0) 36 was utilized to evaluate the association between NLRX1 mRNA expression and both tumor purity and the infiltration of immune cells in the TCGA PRAD dataset using Quantiseq. The analysis included eight immune cell populations: regulatory T cells (Tregs), myeloid dendritic cells, CD8⁺ T cells, CD4⁺ T cells, M1 and M2 macrophages, B cells, and neutrophils. The CAMOIP 37 online tool was employed to examine the expression association between NLRX1 and some immune regulators, including immune checkpoints (CD276, VTCN1, TNFRSF14, PDCD1, and ICOS), TNFSF8, and CXCL10.

### Transwell invasion assay

Invasion assay was assessed using 24-well Transwells (8 μm, Greiner Bio-One) pre-coated with Matrigel for 2 h. 4 × 10⁴ cells/well in serum-deprived medium were added to the upper chamber; complete medium was in the lower. Post incubation, invading cells were immobilized, stained with 0.2% crystal violet, and quantified at 595 nm, as previously described 38.

### Wound healing migration assay

Cells were plated in 12-well culture inserts (Ibidi, Martinsried, Germany) at 4 × 10⁴ cells per well. After attachment, the inserts were removed and fresh medium added. Cells were pretreated with mitomycin C (3 μg/ml) for 30 min, then incubated with or without AKT inhibitor VIII (10 μM) or U0126 (10 μM). Cell migration was observed by light microscopy (4× objective) and quantified using ImageJ software.

### Annexin V apoptosis assay

Cells were seeded at 1 x 10^5^ cells/well in 6-well plates overnight for attachment, then treated with serum-free media and trypsinized. Cells were stained with Annexin V and PI using the FITC Annexin V Apoptosis Detection Kit (BioLegend) and examined for viability by FACSCalibur flow cytometry, as previously described 38.

### Intracellular ROS production and mitochondrial mass measurements

Intracellular ROS production under complete or serum-free conditions was measured using H_2_DCFDA (5 μM) and MitoSOX (5 μM), which detect cytosolic and mitochondrial ROS, respectively. Mitochondrial mass was measured using MitoTracker Green (200 nM). All fluorescence signals were detected using a FACSCalibur flow cytometer (BD, Franklin Lakes, NJ, USA). DCFDA and MitoTracker Green signals were analyzed in the FL1 channel, whereas MitoSOX fluorescence was analyzed in the FL2 channel. Results were expressed as percentages relative to the control group.

### BrdU-based proliferation assay

A BrdU ELISA kit (Roche) was employed to determine cell proliferation, following the supplier's guidelines, as described previously 38. PC3 and LNCaP cells (2 × 10⁴/well) were seeded in black 96-well plates and serum-starved for 12 h, then cultured for 24 h (PC3) or 48 h (LNCaP) with or without 10% FBS. BrdU labeling reagent was added for 2 h, followed by fixation, anti-BrdU-POD incubation (90 min), and substrate addition. Chemiluminescence was measured after 5 min, with control group values set as 100% proliferation.

### Cell cycle analysis

Cells (3 × 10⁵ per well) were seeded in 6-well plates overnight. The next day, cells were deprived of serum for 24 h, then treated with 10% FBS for 12 or 24 h. Post treatment, cells were trypsinized, centrifuged at 2000 rpm for 6 min, and fixed in 70% ice-cold ethanol overnight at 4°C. The day after, cells were centrifuged again, resuspended in 500 μl of the cell cycle analysis kit (BD Biosciences), and then analyzed by flow cytometry (BD FACSCalibur).

### Quantitative polymerase chain reaction (Q-PCR)

Cells were harvested using Trizol (Roche Diagnostics), and RNA extraction was performed following the manufacturer's guidelines. cDNA was generated from 1-2 µg of RNA with a reverse transcription kit (Promega). Q-PCR used SYBR Green Master (Roche Diagnostics) in 96-well plates using the ABI QuantStudio 5 (Applied Biosystems), as described previously 39. The PCR primers are listed in **Table 1.**

### Immunoblotting

Cells were collected in RIPA buffer, sonicated, and heated at 98 °C. Protein concentration was measured (Bio-Rad). The lysates underwent separation via SDS-PAGE, were transferred to PVDF membranes, blocked with milk in TBST, incubated with primary and HRP-conjugated secondary antibodies, and visualized using ECL on the ChemiDoc™ MP system. Densitometric analysis of Western blot bands was performed using ImageJ.

### Immunoprecipitation

To investigate NLRX1-AKT protein interaction, stimulated cells were lysed in RIPA buffer with 150 mM NaCl. Following centrifugation at 4 °C, we precleared supernatants by incubating with normal IgG and protein A-agarose beads for 30 min. Supernatants underwent antibody-mediated immunoprecipitation using 0.5 µg of primary antibody overnight, followed by 1 h rotation with protein A-agarose beads at 4 °C. We washed co-immunoprecipitated complexes with RIPA buffer supplemented with 300 mM and 150 mM NaCl. After boiling beads in the sample loading buffer and centrifuging, we conducted SDS-PAGE and Western blotting analysis.

### Confocal microscopy

Cells were incubated with Mitotracker Red (100 nM) for 60 min before fixation with 4% paraformaldehyde for 15 min. After washing with PBS, cells were permeabilized with 0.2% Triton X-100 in PBS for 20 min and blocked with 5% BSA containing normal IgG (1:300) for 1 h. Cells were then incubated overnight at 4 °C with primary antibodies diluted in 1% BSA. After washing the cells were immunostained with fluorophore-conjugated secondary antibodies for 1 h in the dark. Lastly, nuclei were counterstained with DAPI, and coverslips were mounted. Images were acquired using a 63× Plan-Neofluar oil immersion objective on an LSM 780 confocal microscope.

### Generation of DNA constructs and protein interaction mapping

The construction of the DNA plasmid encoding the full-length (aa 1-975) of the human NLRX1 coding sequence was achieved by PCR amplification with primers nlrx1-BamHI-F1 and nlrx1-XhoI-R2 (which introduced BamHI and XhoI sites). A similar approach was used to amplify the truncated NLRX1 coding sequence encoding aa 76 to 975 (deletion of the MTS domain), aa 1 to 556 (deletion of the LRR domain), and aa 556 to 975 (LRR domain only) using primer pairs nlrx1-BamHI-F2+nlrx1-XhoI-R2 and nlrx1-BamHI-F1+nlrx1-XhoI-R3, respectively. The PCR products were ligated into the BamHI and XhoI sites of the Myc-tagged pJCR vector to express full-length and fragmented constructs of Myc-tagged NLRX1 proteins. The primer sequences for constructing NLRX1 plasmids are listed in** Table 2**. The plasmids for expression of the full-length HA-tagged AKT protein (Plasmid #73408), and truncated HA-tagged AKT constructs encompassing amino acids 1-149 (Plasmid #73410), 120-433 (Plasmid #73411), and 1-408 (Plasmid #73412) were procured from Addgene (Watertown, USA).

### NLRX1-AKT Modeling and MD Simulation

To investigate the interaction between NLRX1 and AKT, AlphaFold-Multimer 40 was used to build the model, which was then subjected to 100 ns molecular dynamics (MD) simulations in GROMACS 41 with the CHARMM36 force field 42. Post-simulation, the complex stability and interaction dynamics were analyzed by computing the root-mean-square deviation (RMSD) and intermolecular hydrogen bonds over time using gmx rms and gmx hbond, respectively. RMSD-based clustering was performed using gmx cluster, and the top-clustered coordinate was used for interaction analysis. Residue-level interactions were examined using LigPlot+ 43 and visualized using ChimeraX 44. Two-dimensional plots were generated using Grace v5.1.21 (http://plasma-gate.weizmann.ac.il/Grace/).

### Animal xenograft model, cryosectioning, and immunofluorescence staining

Six-week-old male BALB/c nude mice (CAnN.Cg-Foxn1nu/CrlNarl) were obtained from the National Center for Biomodels, National Institutes of Applied Research (Taipei, Taiwan). To establish the xenograft model, 1 × 10⁷ PC3 cells stably expressing either shCTL or shNLRX1 were suspended in 100 μl PBS and subcutaneously injected into the posterior flanks of each mouse. Four weeks after inoculation, mice were sacrificed, and tumors were excised and weighed. All procedures were performed according to protocols approved by the Institutional Animal Care and Use Committee of the College of Medicine, National Taiwan University (Approval No. 20230054). Tumor samples were fixed in 4% paraformaldehyde, cryoprotected in 30% sucrose, embedded in OCT compound, and sectioned at a thickness of 20 μm using a cryostat. For immunofluorescence staining, sections were incubated overnight at 4 °C with a primary antibody against Ki-67 (Abcam, ab16680; 1:100 dilution). After washing, sections were incubated with Alexa Fluor 488-conjugated secondary antibody (1:500 dilution) for 1 h at room temperature. Fluorescence images were captured using a fluorescence microscope under identical exposure conditions to ensure consistency for quantitative analysis.

### Statistical analysis

Statistical analyses were executed using GraphPad Prism 8 software (San Diego, CA, USA). To make comparisons, the 2-tailed Student's t-test was employed, and the results were stated as the mean ± SEM. A Kaplan-Meier Survival analysis, along with a log-rank significance test, was employed to assess survival differences among the two groups. For the TCGA gene expression data, the Mann-Whitney test was used for non-parametric data. A p-value < 0.05 was deemed significant.

## Results

### NLRX1 is upregulated in PCa tissues

**Figure 1:**
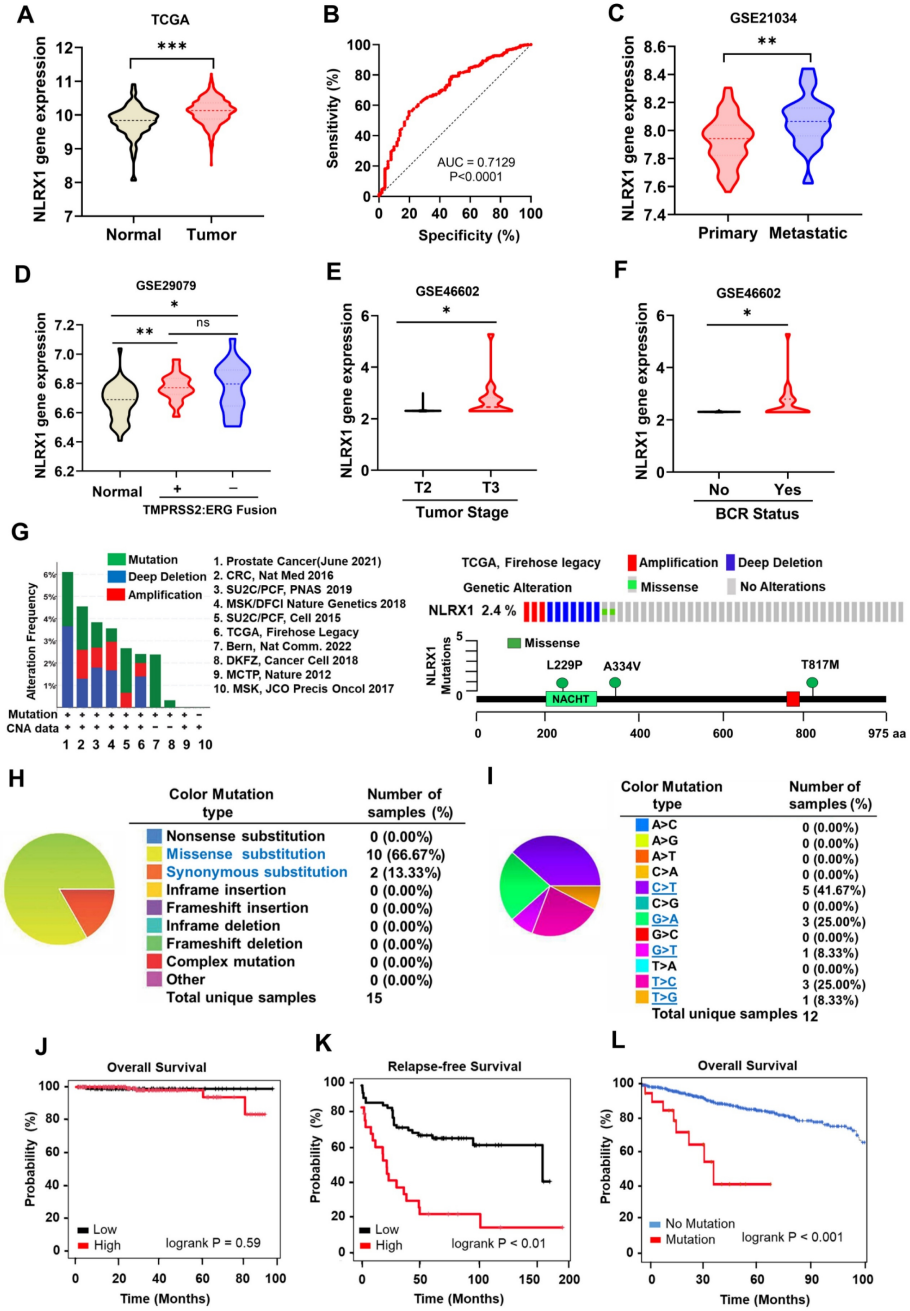
** Elevated NLRX1 expression in PCa tissues. (A)** The TCGA-PRAD database was used to examine NLRX1 mRNA expression levels in PCa tissues. **(B)** ROC curve was generated using the TCGA-PRAD database to differentiate between prostate tumor tissues and normal tissue groups based on NLRX1 gene expression. **(C)** Using the GEO database GSE21034, NLRX1 mRNA expression in both primary and metastatic PCa tissues was assessed. **(D, E, F)** The GEO databases facilitated the analysis of the correlation between NLRX1 expression and ERG, TMPRSS2 fusion status (D, from GSE29079), tumor stage (E, from GSE46602), and biochemical recurrence (BCR) (F, from GSE44602). **(G)** The cBioPortal web browser was employed to investigate the frequency of alterations in the NLRX1 gene across various PCa studies (left panel), and a lollipop plot was used to illustrate mutations in the NLRX1 protein sequence (right panel). **(H, I)** The COSMIC database provided an overview of the types of mutations (H) and substitution mutation types of NLRX1 in PCa (I). **(J, K, L)** The TCGA datasets of PCa patients and the Kaplan-Meier plotter were used to analyze overall survival (OS) (J), relapse-free survival (RFS) (K, from GSE54460), and OS stratified by NLRX1 mutation status (L). *p < 0.05, **p <0.01, ***p <0.001, ns, not significant.

To obtain insights into the correlation between NLRX1 expression and the development of PCa, we initially conducted a bioinformatic analysis utilizing the accessible TCGA-PRAD dataset, which was primarily acquired from UCSC XENA. We compared NLRX1 expression from the GTEx for normal prostate tissue (n = 101 healthy humans) and TCGA for prostate adenocarcinoma (n = 495 PCa patients). The analysis data revealed that the NLRX1 gene was elevated in tumor samples compared to healthy tissues (**Fig. 1A**). To evaluate the effectiveness of NLRX1 gene expression in discriminating between tumor and normal prostate tissues, we employed receiver operating characteristic (ROC) curve analysis. The results showed that NLRX1 displayed a substantial area under the curve (AUC) of 0.7129 in the TCGA-PRAD mRNA data, indicating its potential as a diagnostic marker (**Fig. 1B**). Additional comprehensive analyses revealed that NLRX1 expression was markedly elevated in metastatic PCa tissues relative to primary PCa tissues (**Fig. 1C**). Furthermore, we scrutinized patients with and without TMPRSS2-ERG fusion. The results indicated that the presence or lack of TMPRSS2-ERG fusion was unrelated to NLRX1 expression in PCa tissues (**Fig. 1D**). Notably, clinical data analysis demonstrated a significant association between elevated NLRX1 expression and advanced tumor stages (**Fig. 1E**) and biochemical recurrence (BCR) (**Fig. 1F**) in PCa patients, indicating a positive correlation between NLRX1 level and an unfavorable clinical prognosis. We took the opportunity to explore the various types of alterations and the mutation frequency of the NLRX1 gene by utilizing the cBioPortal database. Analysis revealed 3 types of alterations, including mutation, deep depletion, and amplification of the NLRX1 gene in ten PCa datasets (**Fig. 1G, left panel**). Using the TCGA-Firehose legacy dataset containing 492 samples, the alteration frequency of NLRX1 was 2.4% in PCa, comprising amplification in three cases, deep deletion in seven cases, and missense mutation in two cases (**Fig. 1G, right upper panel**). The mutation landscapes provided further insights into the types, sites, and number of cases involving modifications to the NLRX1 gene (**Fig. 1G, right lower panel**). Moreover, we evaluated the mutation types of NLRX1 using the COSMIC database. As illustrated in the pie chart, out of 15 samples with NLRX1 mutation, 10 samples had missense substitutions (66.67%), and 2 samples had synonymous substitutions (13.33%) (**Fig. 1H**). The substitution mutations mainly included C > T (41%), G > A (25%), T > C (25.00%), G > T (8%), and T > G (8%) (**Fig. 1I**). Following this, we explored whether NLRX1 expression affects the prognosis of PRAD patients. Analysis of TCGA datasets exhibited that NLRX1 expression was not significantly associated with unfavorable survival outcomes (**Fig. 1J**). However, the analysis from GSE54460 revealed that NLRX1 was associated with relapse-free survival (**Fig. 1K**). From the TCGA dataset via cBioPortal, we also found that patients with NLRX1 mutation displayed lower overall survival than patients with wild-type NLRX1 (**Fig. 1L**). Moreover, when analyzing NLRX1 gene expression in benign prostate hyperplasia (BPH), the data revealed no significant change in NLRX1 in normal subjects and BPH patients (**Fig. S1A**), while it was increased in PCa tissues compared to that in BPH patients (**Fig. S1B**). These findings imply that NLRX1 is a prognostic biomarker of PCa and is associated with an adverse prognosis for PCa patients.

### Association between NLRX1 expression and immune cell profiles in human PCa tissues

**Figure 2:**
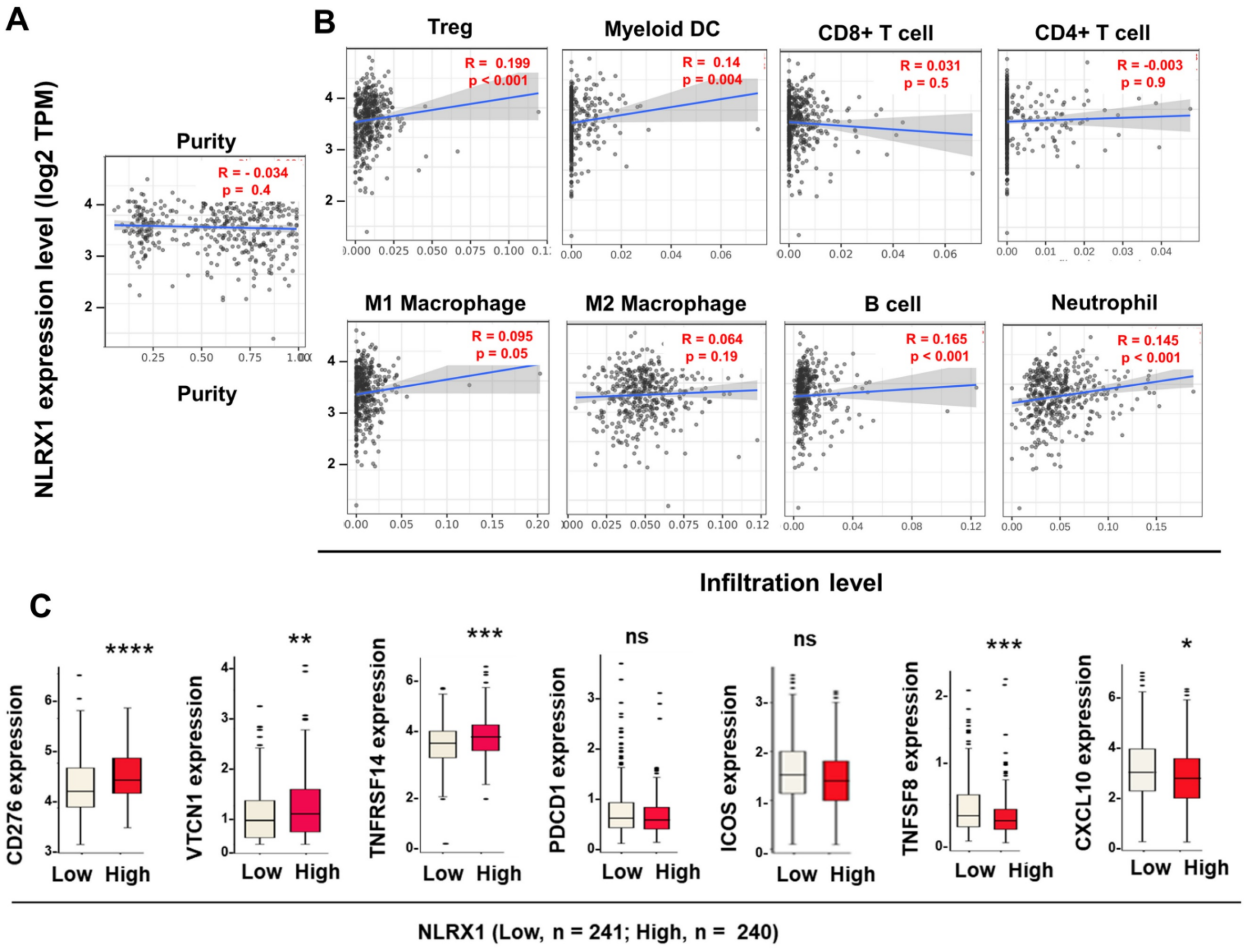
** Correlations between NLRX1 expression and immune infiltration and immune-checkpoint blockades (ICB)-relevant genes in PCa. (A, B)** The TIMER tool was used to assess the correlation between NLRX1 expression and tumor purity (A), as well as the infiltration levels of 8 immune cell types (B). Pearson correlation coefficients (R) and *P*-values are shown. **(C)** The CAMOIP tool was utilized to assess the differences in mRNA expression of ICB-related genes (CD276, VTCN1, TNFRSF14, PDCD1, ICOS, TNFRSF8, and CXCL10) between high- and low-NLRX1 groups within the TCGA-PRAD cohort. *P < 0.05; **P < 0.01; ***P < 0.001; ns, not significant.

Given the critical role of immune cell infiltration in tumor development, progression, and prognosis 45,46, we examined the association between NLRX1 expression, tumor purity, and immune cell infiltration in PCa. Next, we assessed the correlation between NLRX1 expression, tumor purity levels, and immune invasion levels by quanTIseq using the TIMER2 tool. Our results showed that NLRX1 expression was not correlated to tumor purity (**Fig. 2A**). In contrast, it was positively correlated with the infiltration levels of Tregs, myeloid DCs, neutrophils, and B cells, weakly correlated with M1 macrophages, and of no significant relation with CD4^+^ T cells, CD8^+^ T cells and M2 macrophages (**Fig. 2B**). Concurrently, we found that elevated NLRX1 expression was associated with the expression of immune checkpoint-relevant genes in different manners. NLRX1 level was positively correlated to CD276, VTCN1, and TNFRSF14 (CD270), without affecting PDCD1 or ICOS. In addition, NLRX1 expression is negatively associated with other immune regulators such as TNFSF8 and CXCL10 (**Fig. 2C**). Collectively, these findings indicate a possible correlation between NLRX1 expression and a pro-PCa immune environment, supporting a potential pro-oncogenic role of NLRX1 and suggesting its relevance in modulating immunotherapeutic responses.

### Serum-free conditions upregulate NLRX1 expression in PCa cells via gene transcription and protein stabilization

**Figure 3:**
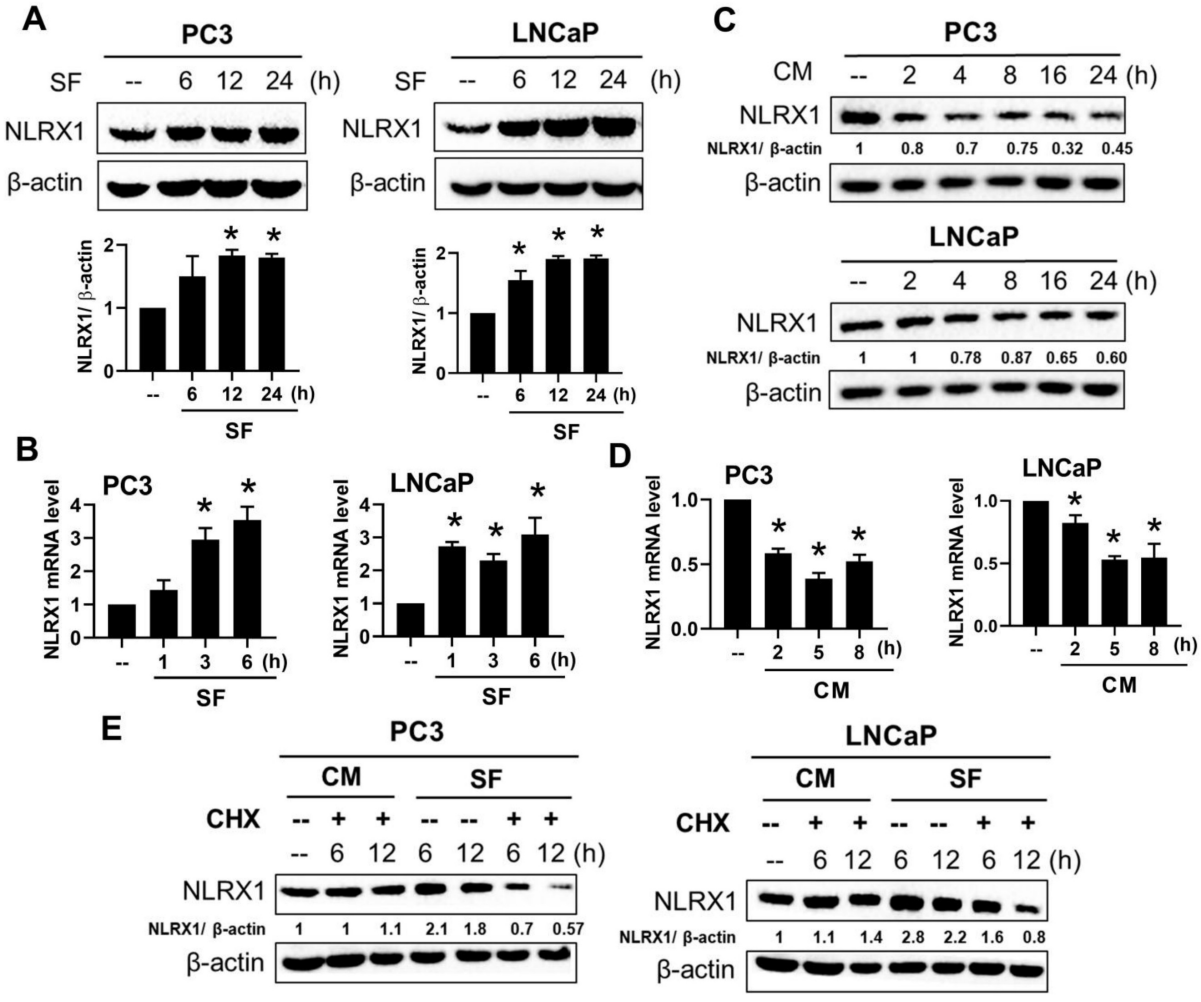
** Serum starvation enhances NLRX1 expression through transcriptional activation in PCa cells. (A, B)** PC3 and LNCaP cells were cultured in serum-free (SF) conditions for the indicated times. NLRX1 protein and mRNA levels were analyzed by immunoblotting (A) and qRT-PCR (B), respectively. **(C, D)** After 24 h serum starvation, cells were re-fed with complete medium (CM) for the indicated times. NLRX1 protein (C) and mRNA (D) levels were assessed. **(E)** Cells were treated with SF or CM ± cycloheximide (CHX, 10 μg/mL) for the indicated durations, and NLRX1 protein levels were measured. Data are presented as the mean ± S.E.M. from independent experiments. Densitometric analysis of Western blot bands was performed using ImageJ. NLRX1 intensity was normalized to β-actin, and values were expressed relative to the 0 h control (set to 1). *, p < 0.05 indicates a significant effect of SF or CM in both PC3 and LNCaP cells.

Solid tumors often exist in a poorly vascularized state, resulting in reduced serum and nutrient availability. Although the relevance of serum starvation to *in vivo* tumor biology remains debated 47, it is widely used to mimic nutrient-restricted conditions that cancer cells may encounter within the tumor microenvironment. Because serum contains multiple cytokines and growth factors that can influence basal signaling activity 48, serum deprivation allows assessment of cellular behavior and signaling responses independent of exogenous growth stimulation 49. We aimed to investigate how NLRX1 is regulated in this serum-deprived context. Accordingly, we examined the effects of NLRX1 expression on cell survival and related phenotypes under serum-deprived conditions. We found that serum-free conditions increased NLRX1 protein expression in both PC3 and LNCaP cells within 6-24 h (**Fig. 3A**). Accordingly, serum-free conditions increased the mRNA level of NLRX1 in PC3 and LNCaP cells (**Fig. 3B**). Vice versa, we found serum administration in pre-starved PC3 and LNCaP cells time-dependently decreased NLRX1 protein (**Fig. 3C**) and gene expression (**Fig. 3D**), indicating that serum is a negative regulator of NLRX1 mRNA expression. Despite this finding, we still wondered if the NLRX1 protein stability might be affected by serum.

Therefore, we compared NLRX1 protein stability under cycloheximide (CHX, 100 µg/ml) treatment in the complete medium or the serum-free medium at different times. We found that CHX dramatically reduced NLRX1 protein expression in both PC3 and LNCaP cells in serum-deprived conditions for 6 h, but not in complete medium conditions for 12 h (**Fig. 3E**). We ruled out the possibility that this change is due to an altered cell viability under serum-free conditions or CHX treatment, as assessed by Annexin V/PI staining (data not shown). Therefore, this finding further indicates that the protein stability of NLRX1 can be maintained by serum.

### NLRX1 silencing inhibits the proliferation of PC3 cells but not LNCaP cells

**Figure 4:**
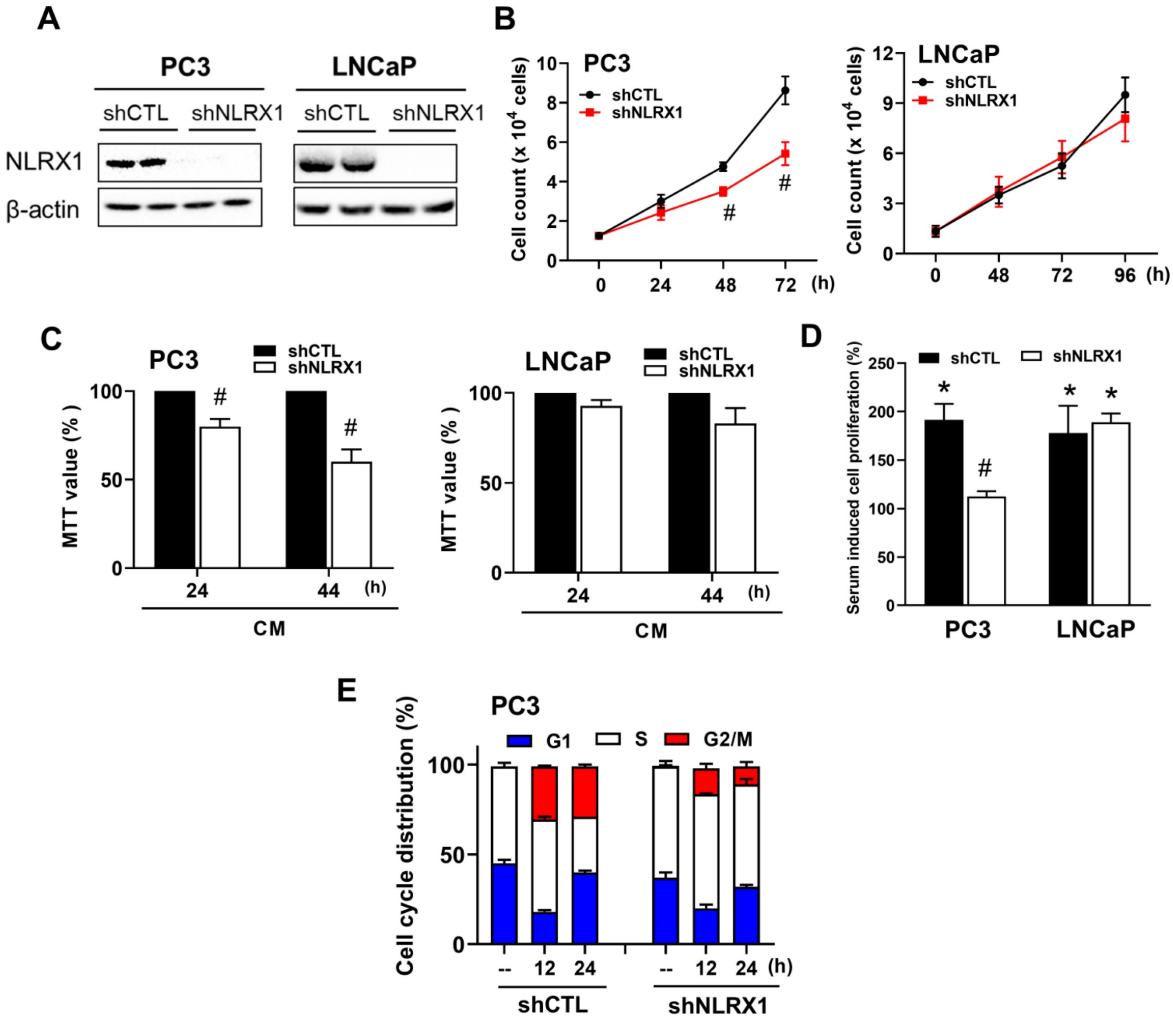
** NLRX1 silencing attenuates cell growth *in vitro* in PC3 cells. (A)** Stable NLRX1-silenced PC3 and LNCaP cell lines were established using a lentiviral short hairpin RNA (shRNA) approach. **(B)** PC3 and LNCaP cells were serum-starved overnight and subsequently stimulated with complete medium (CM) for the indicated time points. Cells were then trypsinized, stained with trypan blue, and counted using a hemocytometer. **(C)** Following overnight serum starvation, PC3 and LNCaP cells were cultured in CM for 24 or 44 h, respectively, after which an MTT assay was conducted. **(D)** After overnight serum starvation, cells were incubated in CM for 24 h (PC3) or 36 h (LNCaP), followed by adding BrdU labeling solution for an additional 2 h. **(E)** PC3 cells were serum-starved for 24 h and stimulated with CM for 12 or 24 h. Following RNAase treatment and propidium iodide (PI) staining, cell cycle distribution was analyzed by flow cytometry. Statistical significance is indicated as follows: **#**p < 0.05, represents a significant effect of NLRX1 silencing compared to shCTL cells; *p < 0.05, indicates a significant effect of CM stimulation compared to serum-starved control cells.

Next, we determined the role of NLRX1 in PCa cell growth. To this end, we used a lentivirus-based silencing method to knock down NLRX1 in PC3 and LNCaP cells (**Fig. 4A**). Using trypan blue staining and cell counting we found that NLRX1 silencing reduced cell growth within 72 h in PC3 cells, whereas this effect was not seen in LNCaP cells (**Fig. 4B**). In agreement with these findings, the data of MTT assay also showed the reduction of cell growth in PC3 cells, although this effect was not seen in LNCaP cells (**Fig. 4C**). Next, serum-induced cell proliferation was determined by BrdU incorporation in pre-starvation cells. Because of the different cell growth rates in both cell lines, we chose 24 h and 48 h after serum treatment as the endpoints in PC3 and LNCaP cells, respectively. As shown in **Fig. 4D**, BrdU uptake was significantly reduced in shNLRX1 PC3 cells, whereas it remained unchanged in shNLRX1 LNCaP cells, indicating that NLRX1 can positively regulate the growth of PC3 cells. Flow cytometric cell cycle analysis of shNLRX1 cells showed a decreased cell population at the G2/M phase, accompanied by an increased cell population at the S phase, in shNLRX1 PC3 cells compared to the control group (**Fig. 4E**). These findings indicate that NLRX1 acts as a positive regulator of cell growth in PC3 cells, and its suppression can lead to cell cycle arrest during the S phase.

### NLRX1 silencing enhances serum-free-induced PCa cell apoptosis independent of ROS levels and autophagy

**Figure 5:**
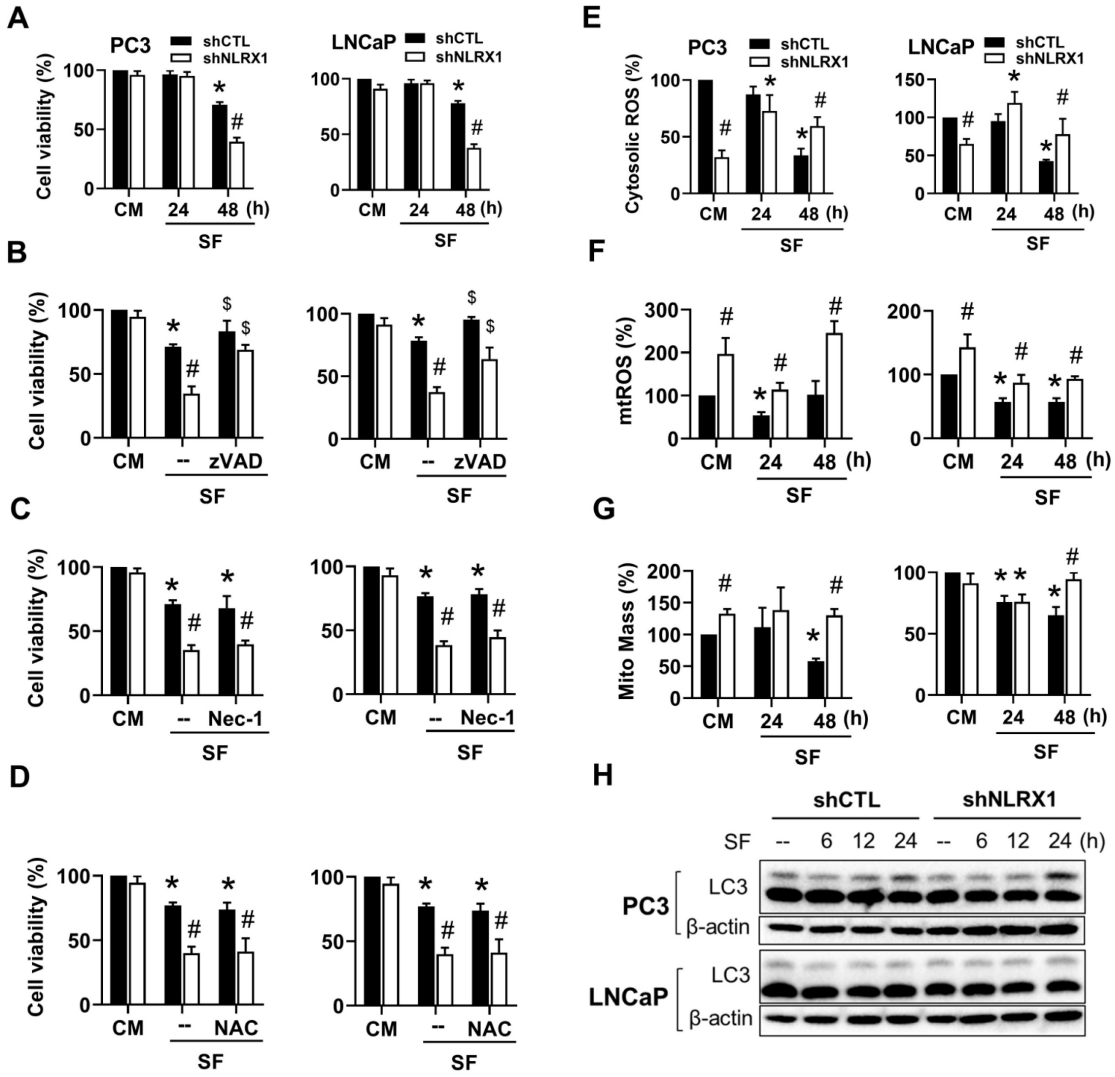
** NLRX1 silencing enhances serum-free induced PCa cell apoptosis independent of ROS levels and autophagy. (A)** PC3 and LNCaP cells were stimulated with SF for the indicated time points. Cell viability was determined by Annexin V-FITC/PI staining using FACS Calibur. **(B, C, D)** Cells were treated with SF alone or co-treated with zVAD-FMK (20 µM), Necrostatin-1 (Nec-1, 10 µM), or N-acetylcysteine (NAC, 5 mM) for 48 h. Cell viability was determined by Annexin V-FITC/PI staining. **(E, F, G)** Cells were treated with SF for the indicated durations, after which cellular ROS levels were measured using DCFDA staining (E), mitochondrial ROS levels were assessed using MitoSOX staining (F), and mitochondrial mass was evaluated using MitoTracker staining(G) in both PC3 and LNCaP cells. **(H)** PC3 and LNCaP cells were stimulated to serum-free (SF) conditions for the indicated durations. Total cell lysates were examined via immunoblotting to assess LC3 and β-actin levels. Data were mean ± S.E.M. in independent experiments. *, p < 0.05 indicates a significant effect of SF compared to the untreated control group. **#**, p < 0.05 indicates a significant effect of shNLRX1 compared to shCTL. $, p < 0.05 indicates a significant protective effect of the inhibitor against SF-induced cell death.

In addition to decreased cell growth in PC3 cells, we would like to explore the significance of NLRX1 in PCa cell viability under serum-free culture, especially when NLRX1 expression is increased. We found that serum-free culture led to a slight decrease in cell viability in both PCa after 48 h, and this effect was enhanced by shNLRX1 (**Fig. 5A**). Further using pharmacological inhibitors of apoptosis (zVAD) and necrosis (necrostatin-1, Nec-1), our data indicated that zVAD (20 μΜ) can reduce cell death under serum-free in control and shNLRX1 PC3 and LNCaP cells (**Fig. 5B**). However, Nec-1 (10 μM) failed to affect the cell death induced by serum-free (**Fig. 5C**). To understand if ROS level is increased and contributes to cell death under serum-free culture, we treated ROS scavenger NAC (5 mM) and found it failed to protect cells (**Fig. 5D**). When conducting DCFDA staining which measures hydroxyl free radical and reactive nitrogen species, our data showed that serum-free decreased fluorescent signals at 48 h in both PC3 and LNCaP cells. Intriguingly, the basal ROS signal was lower in shNLRX1 cells, and, unlike in control cells, serum-free conditions in shNLRX1 cells increased the ROS levels at 24 and 48 h (**Fig. 5E**). When conducting mitoSOX staining to measure mitochondrial O_2_^-^, we found that shNLRX1 increased mtROS levels in both cell lines at the resting state. After changing to a serum-free medium, mtROS levels were decreased at 24 h in shCTL PC3 and LNCaP cells. Moreover, this effect was recovered in PC3 cells but was prolonged for up to 48 h in LNCaP cells. Instead, shNLRX1 cells exhibited higher mtROS levels than shCTL cells, and this effect was still maintained after serum-free treatment (**Fig. 5F**). These data suggest that ROS changes under serum-free conditions might not be involved in cell death in control and shNLRX1 cells, despite the higher mtROS levels in serum-free-treated shNLRX1 cells. NLRX1 is a mitophagy sensor, and mitophagy ensures the clearance of damaged or dysfunctional mitochondria 50. Thus, NLRX1-dependent mitophagy alleviates ischemic injury and promotes cancer progression 51. We wonder if the mtROS increase in serum-free cultured shNLRX1 cells results from the impaired mitophagy. To this end, we measured mitochondrial mass using Mitotracker staining. We found that serum-free conditions gradually decreased mitochondrial mass in both cell lines within 48 h, while NLRX1 silencing reversed this effect (**Fig. 5G**). Next, we determined the effect of serum-free conditions on autophagy. We found that both PCa cells displayed high LC3II levels at the resting state, and serum-free conditions did not affect the LC3II level within 24 h, either in shCTL or shNLRX1 cells (**Fig. 5H**), suggesting that autophagy/mitophagy might not be involved in serum-free-induced PCa death within 48 h. All these findings suggest that NLRX1 silencing enhances serum-free-induced PCa cell apoptosis independent of ROS levels and autophagy.

### NLRX1 silencing reduces cell migration and invasion in PCa cells

**Figure 6:**
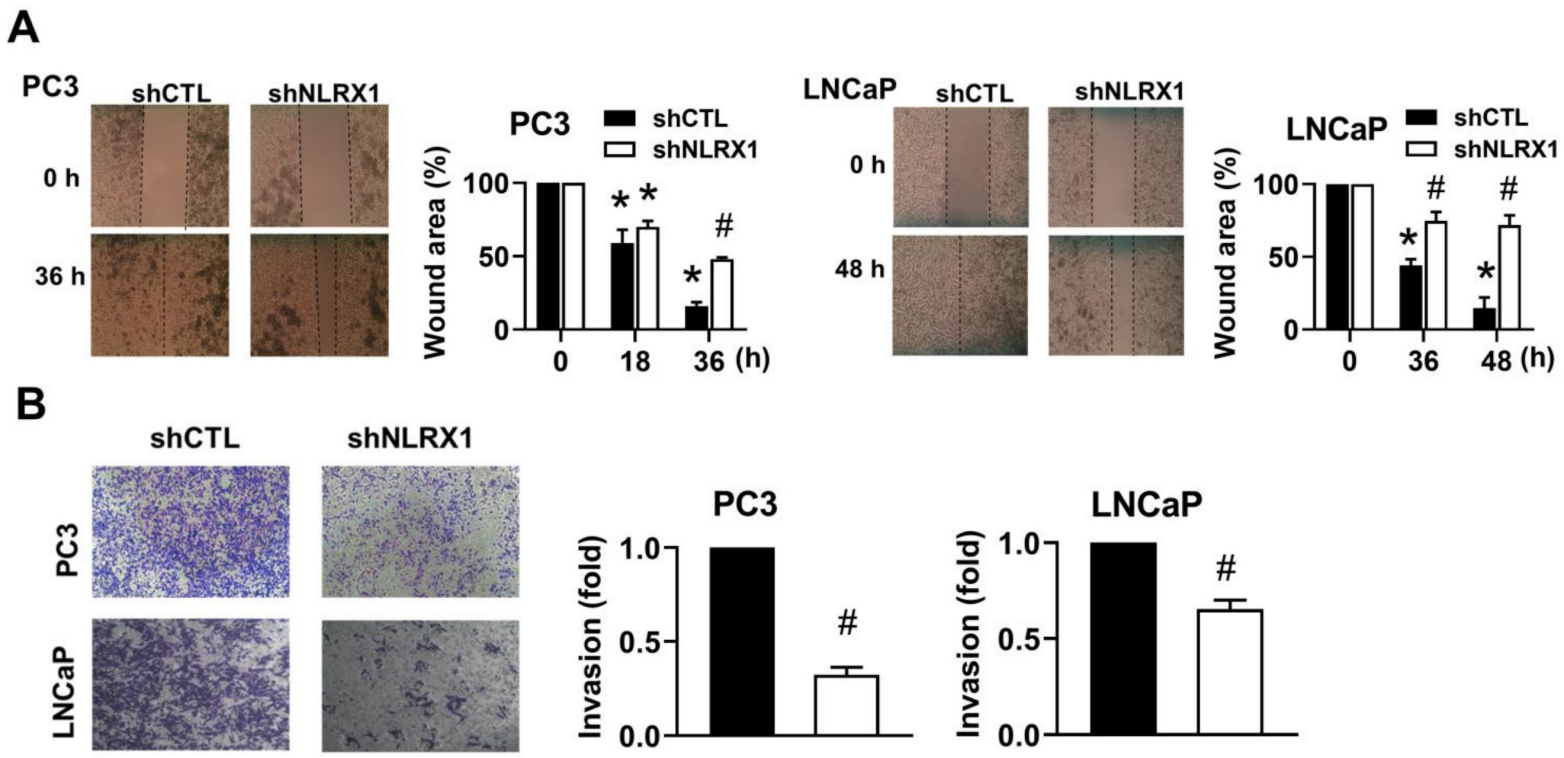
** NLRX1 silencing attenuates the migration and invasion ability of both PC3 and LNCaP cells. (A)** PC3 and LNCaP cells were pre-treated with mitomycin C (1 μg/ml) for 30 min to block proliferation, then subjected to a wound healing assay. Images were taken at 0 h and indicated time points, and wound closure was quantified. **(B)** Cells were seeded in Matrigel-coated Boyden chambers with serum-free medium in the upper chamber and complete medium in the lower chamber. After 28 h, invasive cells were stained, imaged, and quantified. Data were mean ± S.E.M. in independent experiments. *, p < 0.05 indicates a significant difference compared to the untreated control group. **#**, p < 0.05 indicates a significant effect of shNLRX1 compared to shCTL.

Besides regulating cell growth and viability, we further determined the role of NLRX1 in PCa cell migration and invasion. We used the scratch wound healing method for determining cell migration. The findings indicate that shNLRX1 inhibited migration in both PC3 and LNCaP (**Fig. 6A**). We further performed a Matrigel-coated Boyden chamber assay to determine cell invasion. We found that shNLRX1 significantly reduced the invasion abilities of both PC3 and LNCaP cells (**Fig. 6B**).

### NLRX1 silencing reduces AKT and ERK activation in serum-free conditions

**Figure 7:**
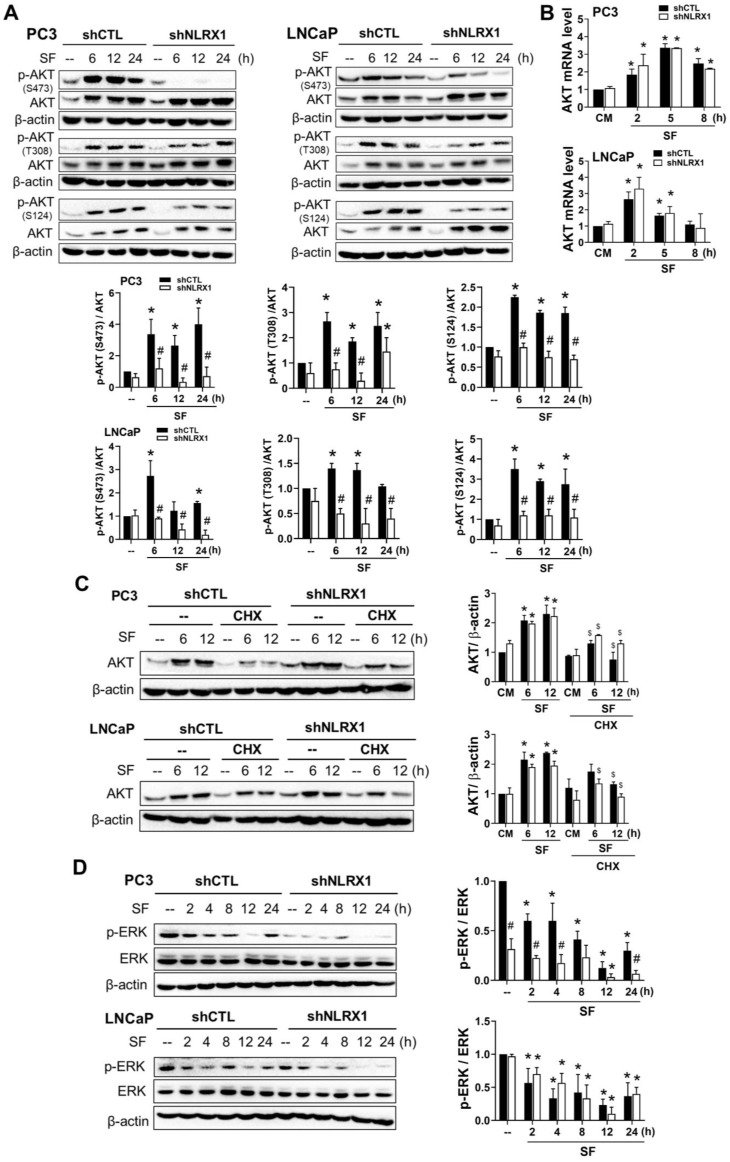
** Differential regulation of AKT and ERK phosphorylation by NLRX1 silencing under serum-free conditions. (A)** PC3 and LNCaP cells were cultured in serum-free (SF) conditions for the indicated times. Immunoblotting was used to detect p-AKT (S473, T308, S124), total AKT, and β-actin. **(B)** AKT mRNA levels under SF were measured by qRT-PCR. **(C)** Cells were treated with SF ± cycloheximide (CHX, 10 μg/ml) for the indicated times; AKT protein levels were analyzed and normalized to β-actin. **(D)** p-ERK and β-actin levels were assessed under SF by immunoblotting. Densitometric analysis of Western blot bands was performed usingImageJ. Protein expression was normalized to β-actin, and values were expressed relative to the 0 h control (set to1). Data are mean ± S.E.M. from independent experiments. *, p < 0.05 indicates a significant effect of SF compared to the untreated control group. **#**, p < 0.05 indicates a significant effect of shNLRX1 compared to shCTL. $, p < 0.05 indicates a significant effect of CHX.

To understand the molecular mechanisms underlying the roles of NLRX1 in regulating cell growth, viability, migration, and invasion under serum-free conditions, we determined AKT and ERK signaling pathways. In agreement with previous findings showing that serum-free media induce AKT activation 52-54, we also observed this event in PC3 and LNCaP cells. Of note, AKT protein level is accordingly increased (**Fig. 7A**). In addition, AKT protein induction by serum-free media is dependent on gene expression (**Fig. 7B**), and NLRX1 silencing did not affect the mRNA (**Fig. 7B**) and protein (**Fig. 7A**) levels of AKT. In addition to analyzing mRNA levels, we treated cells with cycloheximide (100 μg/mL) to assess the role of NLRX1 in AKT protein stability. As shown in **Fig. 7C**, the serum-free-induced AKT protein expression at 6 h and/or 12 h in PC3 and LNCaP cells was significantly reduced, with higher reduction extents in PC3 cells than in LNCaP cells. Moreover, such downregulation extents caused by cycloheximide were not altered in shNLRX1 cells. Taken together, NLRX1 does not affect serum-free-induced AKT gene and protein expression, but can inhibit AKT activation. Moreover, S124 phosphorylation on AKT has been shown to augment its phosphorylation at S473 55. Here, we also found that serum-free conditions can induce S124 phosphorylation, and this effect is also attenuated in shNLRX1 cells (**Fig. 7A**). These findings strengthen the role of NLRX1 in mediating AKT activation caused by serum-free conditions. Besides AKT, we also determined the effect of serum-free conditions on ERK. We found that in both cell lines, serum-free time-dependently decreased ERK phosphorylation within 12 h, and there was a slight rebound increase at 24 h. Unexpectedly, NLRX1 silencing decreased ERK phosphorylation before and after serum-free treatment in PC3 cells but not in LNCaP cells (**Fig. 7D**). To understand how serum-free treatment upregulates AKT expression, we determined the effects of AKTi and U0126. We found that AKTi did not alter the increased AKT protein level nor affect ERK in serum-free conditions in PC3 and LNCaP cells (**Fig. S2**). Similarly, ERK inhibitor U0126 failed to affect AKT expression and activation in serum-free conditions (**Fig. S2**).

### NLRX1 silencing does not affect AKT upstream signaling pathways: PDK1, mTORC2, and PLK4

Previous studies demonstrated that phosphorylation of AKT at S473 in the C-terminal hydrophobic motif by mTORC2 (S2481) and phosphorylation at T308 in the catalytic domain by PDK1 are required for its full activation. Thus, they have been employed as indicators of AKT activation 56. Here, we found that serum-free conditions can upregulate PDK1 and increase PDK1 auto-phosphorylation at S241, and both effects were not altered in shNLRX1 cells (**Fig. S3A**). As to mTOR phosphorylation at S2481 (an index of mTORC2 activation), it was not changed by serum-free conditions in control and shNLRX1 PC3 cells (**Fig. S3A**). These findings suggest that PDK1-AKT but not mTORC2-AKT is involved in serum-free-induced AKT phosphorylation. Apart from the common attention on AKT phosphorylation at S473 and T308 by mTORC2 and PDK1, respectively, PLK4 is another upstream kinase of AKT, but it is less investigated. PLK4 was shown to phosphorylate AKT at S124, T308, and S473, and PLK4-mediated phosphorylation at S124 significantly augments the phosphorylation at S473 55. Here, to fully decipher the underlying mechanisms of attenuating AKT phosphorylation at three sites in the shNLRX1 condition, we determined PLK4. Unlike PDK1 and AKT, the PLK4 protein level was not changed in serum-free conditions in both control and shNLRX1 cells (**Fig. S3B**). Next, we determined the mRNA levels of PDK1 and PLK4 in serum-free conditions. Consistent with the protein levels, PDK1 mRNA levels were time-dependently increased by serum-free conditions to similar extents in control and shNLRX1 cells. In addition, serum-free conditions did not affect PLK4 gene transcription in control and NLRX1-silencing PCa cells (**Fig. S3C**). Next, we are interested in understanding how serum-free conditions increase PDK1 expression. The data from pharmacological inhibitors revealed that neither AKTi nor U0126 can alter the increased PDK1 expression and activation caused by serum-free conditions in both PCa cells (**Fig. S3D**). Taken together, serum-free conditions can transcriptionally upregulate PDK1 expression, and this PDK1 activation is independent of NLRX1, AKT, or ERK.

### NLRX1-dependent AKT and ERK activation contribute to survival and invasion in serum-free conditions

**Figure 8:**
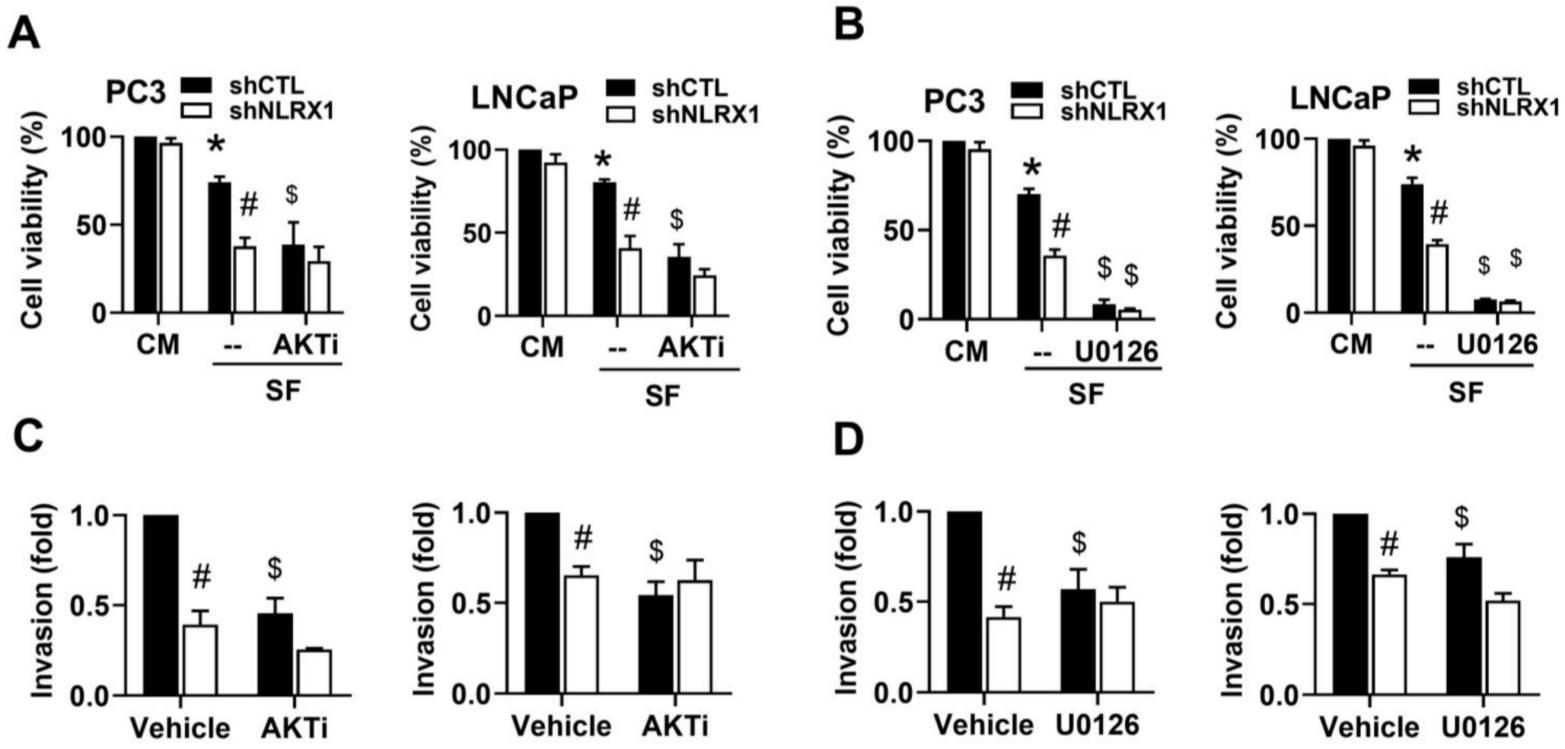
** NLRX1 silencing promotes cell death and inhibits invasion via AKT and ERK suppression under serum-free conditions. (A, B)** Cell viability was measured in control (shCTL) and NLRX1 knockdown (shNLRX1) PC3 and LNCaP cells under complete medium (CM) or serum-free (SF) conditions, with or without AKTi (A) or U0126 (B). **(C, D)** Invasion assays were performed under SF with vehicle, AKTi (C), or U0126 (D). Data are mean ± S.E.M. from independent experiments. *, p < 0.05 indicates a significant effect of SF compared to the untreated control group. **#**, p < 0.05 indicates a significant effect of shNLRX1 compared to shCTL. $, p < 0.05 indicates a significant effect of the inhibitor treatment under SF conditions.

After observing that NLRX1 plays a role in cell survival and invasion in PCa cells under serum-free conditions, we were interested in evaluating the roles of AKT and ERK signal pathways in the cellular function of NLRX1. Using pharmacological AKT inhibitor AKTi (5 µM) and ERK inhibitor U0126 (10 µM), we found that serum-free triggered cell death in PC3 and LNCaP cells was markedly enhanced by AKTi and U0126, with higher efficacy of U0126 than AKTi (**Fig. 8A, 8B**). Moreover, both inhibitors also abolished the invasion ability of PC3 and LNCaP cells to similar extents (**Fig. 8C, 8D**). NLRX1 silencing-induced inhibitory effects on cell survival and invasion were phenocopied by the presence of each kinase inhibitor (**Fig. 8A-8D**). These findings suggest that AKT (in PC3 and LNCaP cells) and ERK (only in PC3 cells) are positively regulated by NLRX1 and contribute to cell survival and invasion in serum-free conditions.

### NLRX1-dependent AKT activation contributes to cell growth and migration in complete medium conditions

Because cell growth and migration were measured in PCa cells cultured in a complete medium, we were also interested in understanding the roles of AKT and ERK in both events regulated by NLRX1. First, we determined how NLRX1 regulates both kinases in complete medium culturing. We found that serum administration into serum-free cultured cells can downregulate AKT and inhibit AKT activity, and both effects were opposite to the effects of serum-free culture. Similar to our observations in serum-free conditions, NLRX1 silencing reduced AKT phosphorylation at S473 without affecting total AKT downregulation caused by serum supplementation (**Fig. S4A, upper panel**). On the other hand, serum transiently induced ERK phosphorylation in both PCa cell lines, and this effect in PC3 but not in LNCaP cells was attenuated by NLRX1 silencing (**Fig. S4A, lower panel**). In addition, consistent with the effect of serum-free on upregulation of PDK1, serum treatment decreased PDK1 expression and activation without affecting PLK4 protein level. Moreover, the effects on increasing PDK1 expression and activation were not changed by shNLRX1 in both PCa cells (**Fig. S4B**). We also found that when serum was added to pre-starving PC3 control cells, AKTi displayed a more sustained inhibitory effect on cell growth than U0126 (**Fig. S4C, S4D, left upper panels**). In shNLRX1 PC3 cells, where cell growth has been reduced, both inhibitory effects of AKTi and U0126 became marginal (**Fig. S4C, S4D, right upper panels**). In control LNCaP cells, AKTi has a weaker inhibition on cell growth compared to that in PC3 cells, and U0126 has no effect (**Fig. S4C, S4D, left lower panels**). In shNLRX1 LNCaP cells, neither inhibitor can change cell growth (**Fig. S4C, S4D, right lower panels**). In addition, we found that only AKTi, but not U0126, can inhibit PC3 and LNCaP cell migration. The inhibitory effect of NLRX1 silencing on cell migration was non-additive to that of AKTi (**Fig. S4E, S4F**). These findings suggest a more pivotal role of AKT than ERK in PCa growth and migration, and NLRX1-dependent enhancements of cell growth and migration are mediated by AKT activation.

### NLRX1 silencing inhibits growth factor-induced AKT and ERK activation

**Figure 9:**
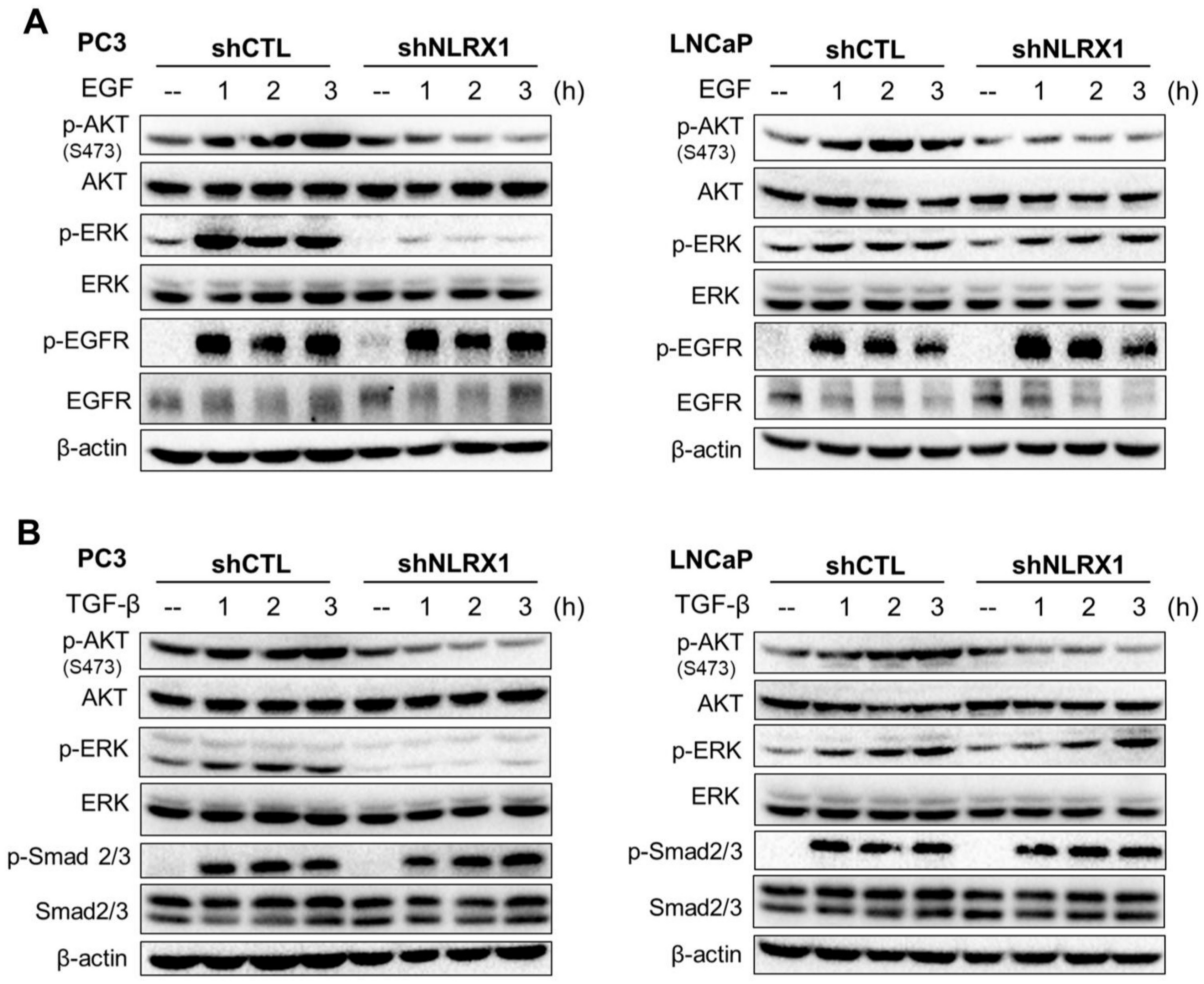
** NLRX1 silencing differentially modulates AKT and ERK phosphorylation in response to EGF and TGF-β. (A)** PC3 cells and LNCaP control (shCTL) or NLRX1-silencing cells (shNLRX1) were treated with EGF for the indicated times. Immunoblots show levels of p-AKT S473, total AKT, p-ERK, total ERK, p-EGFR, total EGFR, and β-actin. **(B)** PC3 and LNCaP shCTL or shNLRX1 cells were stimulated with TGF-β for the indicated times. Blots were probed for p-AKT S473, total AKT, p-ERK, total ERK, p-Smad2/3, total Smad2/3, and β-actin.

Not only regulating AKT and ERK phosphorylation in serum-free medium and complete medium, but we also determined if NLRX1 regulates both growth factor-associated signal pathways upon EGF or TGF-β stimulation. In serum-free cultured cells, we treated cells with EGF (50 ng/ml) or TGF-β (5 ng/ml). We found that shNLRX1 can inhibit AKT and ERK activation caused by both growth factors in PC3 cells, without affecting EGFR-p or Smad2/3-p (**Fig. 9A, B, left panels**). On the other hand, shNLRX1 only inhibits AKT but not ERK in response to growth factors in LNCaP cells (**Fig. 9A, B, right panels**). All these findings indicate that NLRX1 does not affect the upstream signaling pathways of EGF and TGF-β but specifically and positively regulates AKT activity. Its positive regulation of ERK is only in PC3 cells. Such a cell-type-specific role in regulating ERK is similar to what we observe in response to serum, as mentioned above.

### NLRX1 can bind to AKT via LRR and PH domains

**Figure 10:**
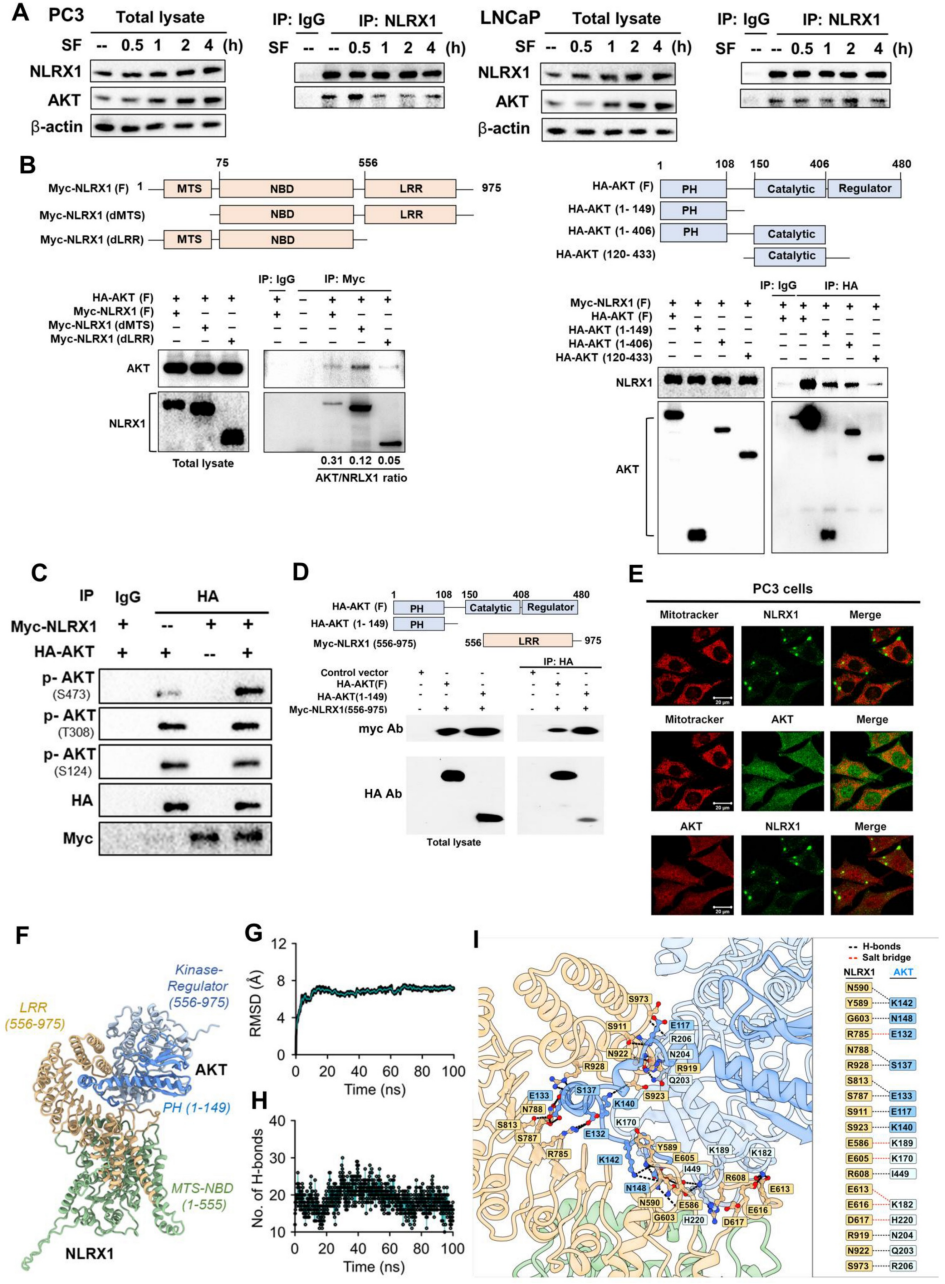
** Binding domains for NLRX1 and AKT interaction. (A)** PC3 and LNCaP cells were cultured in either complete medium (CM) or serum-free medium (SF) following overnight attachment. NLRX1 was immunoprecipitated from cell lysates, and the resulting immunoprecipitates were analyzed by immunoblotting to detect interacting proteins. **(B)** HEK293T cells were co-transfected with: Left, Full-length HA-tagged AKT and various Myc-tagged NLRX1 deletion mutants, or Right, Full-length Myc-tagged NLRX1 and various HA-tagged AKT deletion mutants. After 24 h, cell lysates were immunoprecipitated using anti-Myc or anti-HA antibodies. The immunoprecipitates were then analyzed by immunoblotting with anti-HA or anti-Myc antibodies to determine the regions required for the NLRX1-AKT interaction. Band intensity ratios indicate binding efficiency. **(C)** HEK293T cells were co-transfected with full-length HA-tagged AKT and full-length Myc-tagged NLRX1. After 24 h, co-immunoprecipitation was performed using an anti-HA antibody, and AKT phosphorylation levels were assessed by immunoblotting. (**D**) HEK293T cells were co-transfected with HA-tagged full-length or the AKT PH domain together with a Myc-tagged NLRX1 LRR-domain construct. After 24 h, co-immunoprecipitation was performed using an anti-HA antibody, and the immunoprecipitates were analyzed by immunoblotting with anti-HA and anti-Myc antibodies. **(E)** Confocal microscopy was performed to examine the subcellular localization of AKT and NLRX1, and MitoTracker Red was used to visualize mitochondrial morphology. **(F)** Structural model showing NLRX1 (LRR) interacting with AKT (PH and kinase-regulator domains). **(G)** RMSD plot shows stable NLRX1-AKT interaction over 100 ns MD simulation. **(H)** Hydrogen bond count during MD simulation indicates persistent interactions. **(I)** Residue-level map of H-bonds between NLRX1 and AKT during simulation.

Because NLRX1 silencing attenuates AKT activation without affecting upstream signal pathways regulated by serum, EGF, and TGF-β, we are interested in deciphering the possible protein-protein interaction. Our data of co-immunoprecipitation revealed that NLRX1 can associate with AKT in PC3 and LNCaP cells regardless of culturing in complete medium or serum-free conditions (**Fig. 10A**). Despite both NLRX1 and AKT protein levels being increased in serum-free conditions, we did not observe the increased protein interaction based on the co-immunoprecipitation assay, which might be due to the existence of other mechanisms beyond protein levels. Next, we overexpressed AKT and NLRX1 with different deletion structures and identified their interaction domains. We found that NLRX1 lacking the LRR domain has the weakest interaction with AKT (**Fig. 10B, left panel**). On the other hand, the N-terminal PH domain in AKT is involved in the interaction with NLRX1 (**Fig. 10B, right panel**). When overexpressing Myc-NLRX1 and HA-AKT in HEK293 cells, AKT phosphorylation at S473, but not at T308 or S124, was enhanced (**Fig. 10C**), suggesting that NLRX1 can increase AKT activation via protein-protein interaction. To further confirm the interaction interface between NLRX1 and AKT, we generated an NLRX1 truncation construct spanning amino acids 556-975 (LRR domain only) and co-transfected it with an AKT PH-domain construct for co-immunoprecipitation. The results showed that the NLRX1 LRR domain is sufficient to associate with AKT, and that the interaction with AKT is mediated by the PH domain (**Fig. 10D**). In addition, we examined the subcellular distribution of AKT and NLRX1. Confocal microscopy revealed that in PC3 cells, NLRX1 was predominantly localized in the cytosol, with only a small fraction present on mitochondria, as expected, whereas AKT was distributed throughout the cells. Notably, AKT and NLRX1 exhibited substantial co-localization mainly in the cytosol and mitochondria (**Fig. 10E**).

To elucidate the molecular interaction between AKT and NLRX1, we generated a structural model of the complex (**Fig. 10F**) and assessed its stability through a 100 ns molecular dynamics simulation. As shown in **Fig. 10G**, the RMSD of the AKT-NLRX1 complex indicated its structural stability after ~20 ns. The evolution of intermolecular hydrogen bonds between AKT and NLRX1 is also stabilized after ~20 ns (**Fig. 10H**), indicating strong and persistent associations. Consistent with our suggestion, the LRR domain of NLRX1 was found to interact directly with AKT, through predominantly electrostatic interactions complemented by several hydrophobic contacts. Detailed analysis revealed that the interface between the NLRX1 LRR domain and the AKT PH domain involves ten hydrogen bond pairs, including N590/Y589-K142, G603-N148, N788/R928-S137, S813/S787-E133, S991-E117, S923-K140, and a salt bridge R785-E132 (**Fig. 10I**). In addition, the LRR domain of NLRX1 forms a network of non-covalent interactions with the kinase domain of AKT, comprising nine hydrogen bonds (including those involved in five salt bridges). Notable residue pairs include E586-K189, E605-K170, R608-I449, E613/E616-K182, D617-H220, R919-N204, N922-Q203, and S973-R206.

### NLRX1 knockdown suppresses tumor growth *in vivo*

**Figure 11:**
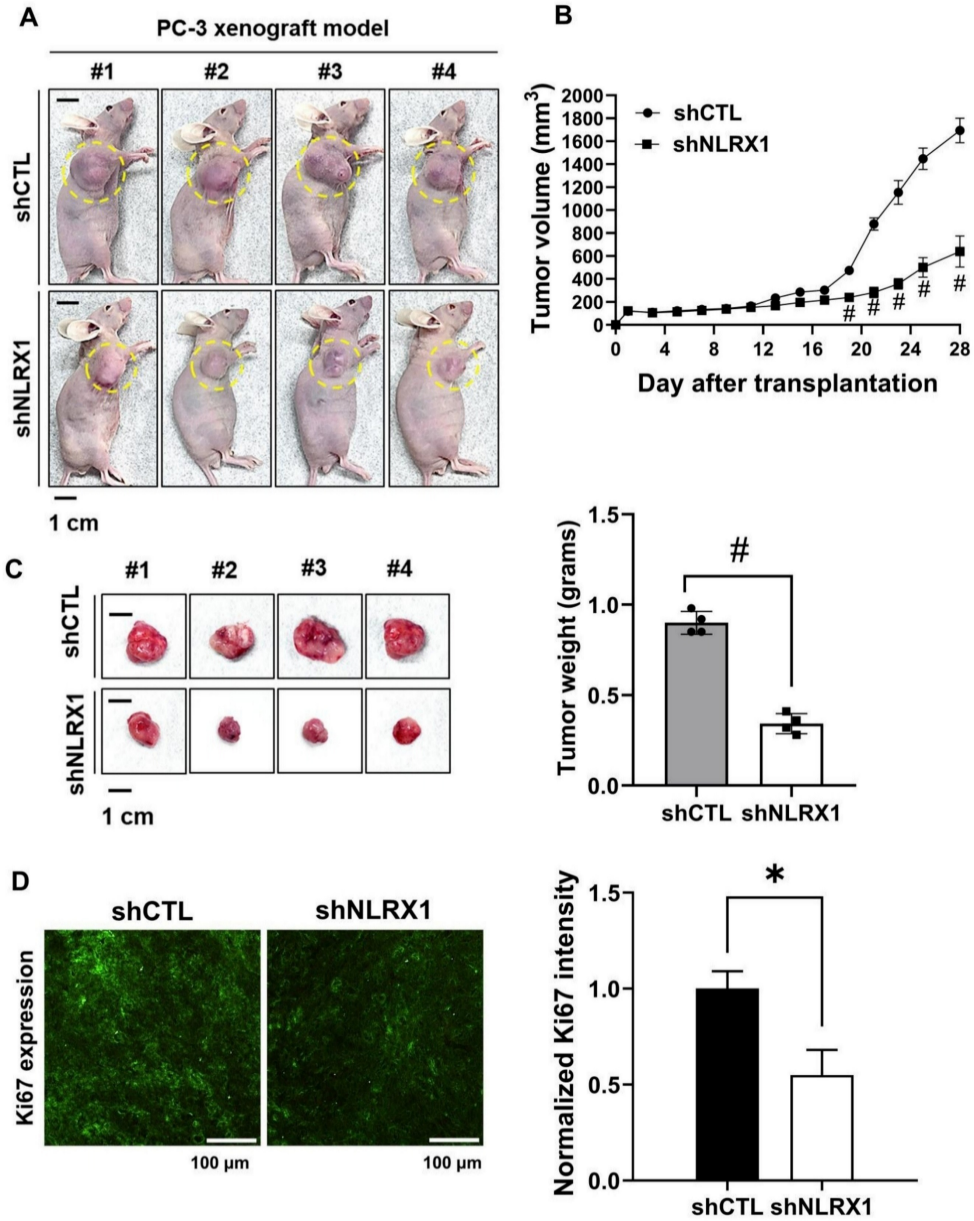
** NLRX1 promotes tumor growth *in vivo*.** shCTL and shNLRX1 PC3 cells (1 × 10^7^ cells) were subcutaneously implanted into male nude mice. The tumors were measured every two days for four weeks. (A) Images of the tumor burden in each group. The yellow circle indicates the tumor region. Scale bar: 1 cm. (B) The tumor volume was calculated as follows: V = 0.5 × (length of the longest diameter) × (length of the shortest diameter)^2^. #, *p*<0.0001. (C) Gross images of xenograft tumors were shown. Scale bar: 1 cm. At the end of the experiment, the tumors were excised and weighed; the bar graph represents the quantification of tumor weights. Data are presented as mean ± S.E.M. #, p < 0.0001. (D) Representative IF staining and quantification of Ki67 in tumor sections from shCTL and shNLRX1 xenografts were shown. Section thickness: 20 μm. Scale bar: 100 μm. Data are presented as mean ± SEM. *, p < 0.05.

To assess the contribution of NLRX1 to tumor development, a xenograft model was generated by subcutaneously implanting PC3 shCTL or PC3 shNLRX1 cells into BALB/c-nu mice. Tumor progression was tracked over time, and representative photographs documenting tumor formation were obtained, with tumor areas marked with yellow circles (**Fig. 11A**). Mice injected with shNLRX1 cells developed markedly smaller tumors than controls, as reflected by reduced tumor volumes (**Fig. 11B**). At the study endpoint, tumors were excised and weighed, revealing a significant decrease in tumor weight in the NLRX1-silenced group (**Fig. 11C**). Immunofluorescence staining showed lower Ki67 expression in tumors lacking NLRX1, suggesting diminished proliferative activity (**Fig. 11D**). Collectively, these results indicate that silencing of NLRX1 restrains PCa growth.

## Discussion

NLRX1 is a multifunctional molecule involved in regulating cellular processes and diseases 7,9,50. Multiple contradictory functions for NLRX1 in cancer have been reported 22. NLRX1 acts as a suppressor in colorectal cancer, hepatoma, gastric cancer, squamous cell carcinoma, histiocytic sarcoma, and pancreatic cancer 24,26-30 or a promoter in mammary cancer cells 33,57. While evidence suggests NLRX1's role in tumorigenesis, studies in PCa are limited. Serum-free and nutrient-deficient conditions are features of solid tumor biology, and many studies commonly use these conditions to explore cancer development. We demonstrate that NLRX1 expression is higher in PCa tissues and promotes PCa cell growth and migration in complete medium, as well as survival and invasion in serum-free conditions through positive regulation of AKT and ERK signaling. NLRX1 may serve as a prognostic biomarker for PCa patients.

In this study, analyses of TCGA and GEO datasets reveal that elevated NLRX1 expression in PCa is associated with unfavorable clinicopathologic features and poor patient outcomes. NLRX1 is upregulated in metastatic disease and is associated with advanced tumor stages, recurrence-related events, and specific genetic alterations. Furthermore, high NLRX1 expression is linked to reduced relapse-free survival and biochemical recurrence, while NLRX1 mutations are associated with poor overall survival in PCa patients. Together, these findings suggest that increased NLRX1 expression in tumors, along with its association with survival pathways, supports a potential role for NLRX1 in PCa progression and tumorigenesis. Our immune profiling further revealed a correlation between NLRX1 expression and tumor immune milieu in PCa. Elevated NLRX1 levels correlate with immunosuppressive cells, including Tregs, myeloid DCs, neutrophils, and B cells. NLRX1 expression was associated with increased CD276 (B7-H3), VTCN1 (B7-H4), and TNFRSF14 (CD270, HVEM), but decreased TNFSF8 (CD30 ligand) and CXCL10. CD276, VTCN1, and TNFRSF14 are immune checkpoint molecules involved in PCa progression 58-61. TNFSF8 induces T cell proliferation 62,63 and promotes PCa survival 64. CXCL10 marks M1 macrophages and inhibits LNCaP cell proliferation 65. Lower TNFSF8 and CXCL10 in high NLRX1-expressing cancer might support tumor formation. Our findings indicate NLRX1's role in promoting immune evasion by enhancing inhibitory signaling and impairing T-cell activation.

The key kinase AKT regulates metabolism, survival, and growth factor signaling 66. In human cancers, the AKT pathway contributes to growth, survival, and tumorigenesis of various cancers, including PCa 67,68. One of our novel findings is the direct interaction between NLRX1 and AKT. Currently, there is no literature elucidating and providing the mechanisms for directly linking NLRX1 to increased AKT signaling. Nevertheless, some indirect association data and controversial findings between NLRX1 and AKT were demonstrated in cancer cells, hearts, and macrophages. NLRX1 ablation has been shown to reduce activation of the mTOR and RISK pathways (including AKT, ERK, and S6K) following ischemia-reperfusion injury in the mouse heart. In addition, increased ischemia-reperfusion injury in NLRX1-deficient hearts is associated with impaired AKT signaling 18,24. In Kupffer cells, NLRX1 expression is downregulated following LPS stimulation, and NLRX1 overexpression promotes a PI3K-AKT-dependent shift of macrophages toward an anti-inflammatory M2 phenotype, indicating that higher NLRX1 expression is associated with enhanced AKT activation 69. Conversely, NLRX1 functions as a tumor suppressor in esophageal squamous cell carcinoma 29 and HCC 31 by inhibiting PI3K/AKT signaling, suppressing cell growth, and epithelial-mesenchymal transition. Despite these opposite findings, the exact mechanisms remain unclear. We demonstrate that NLRX1 positively regulates AKT activation in PCa cells caused by serum-free, EGF, and TGF-β, contributing to cell migration, invasion, proliferation, and viability. Co-immunoprecipitation shows NLRX1 directly binds AKT via its LRR domain, interacting with AKT's PH domain. NLRX1 and AKT overexpression in HEK293T cells confirms NLRX1 as a modulator of AKT signaling, promoting its phosphorylation. Our molecular modeling and dynamics simulations reveal a stable interaction interface. The 100 ns MD simulation and persistent intermolecular hydrogen bonds indicate a strong association. The LRR domain of NLRX1 forms multiple hydrogen bonds and salt bridges with AKT's PH and kinase domains, supporting a robust binding interface. These results establish NLRX1's role in AKT-mediated signaling pathways.

Although serum-free conditions have been shown to activate AKT in PCa cells 52-54 and neuroblastoma cells 70, the mechanisms remain unknown. AKT's complete activation requires phosphorylation at S473 and T308 by mTORC2 and PDK1, respectively 56. Moreover, PLK4 is a favorable therapeutic target in PCa due to its role in promoting the PI3K-AKT pathway and cell migration 66,68. PLK4-dependent phosphorylation of AKT1 at S124 augments AKT1 S473 phosphorylation 55. In this study, we observe that serum-free conditions upregulate PDK1 and AKT gene expression in PC3 and LNCaP cells, without affecting mTORC2 and PLK4 expression. These findings align with reports showing PDK1 upregulation in breast cancer cells 71 and HEK293T cells 72 under serum-free conditions. These changes lead to increased AKT phosphorylation at S473, T308, and S124, and PDK1 auto-phosphorylation at S241. The lower PDK1 mobility in immunoblotting in serum-free conditions might be due to increased phosphorylation 72. We demonstrate that serum-free-induced expression of PDK1 and AKT is independent of NLRX1, AKT, or ERK. In this aspect, we suggest that AMPK might be involved in AKT and PDK1 upregulation, as reported 71. Moreover, while silencing NLRX1 does not affect PDK1 activation, it attenuates AKT activation through direct interaction and modulation of phosphorylation, as we discussed above.

Interestingly, we also observe that in PC3 cells, NLRX1 is predominantly localized in the cytosol, while only a small fraction is present on mitochondria. Although NLRX1 is initially recognized to be localized in the mitochondria, some studies demonstrate that it can be moved between the cytosol and the mitochondria 8,73. AKT is localized in the mitochondria and nuclei. The co-localization of AKT and NLRX1 in the mitochondria might be involved in regulating mitochondrial homeostasis and energy metabolism. As reported, NLRX1 is not only a mitophagy sensor 11 but also a negative regulator of mitochondrial respiration 18,23,74. Similarly, AKT can suppress mitochondrial biogenesis by downregulating PGC-1α (a master regulator of mitochondrial biogenesis) and inhibiting FOXO transcription factors, which normally promote mitochondrial biogenesis and oxidative metabolism. Moreover, AKT shifts cellular metabolism toward glycolysis and inhibits mitochondrial respiration 75. We speculate that mitochondrial loss promoted by the NLRX1-AKT axis might conserve energy under low-nutrient conditions, i.e., serum-free conditions.

Besides regulating AKT in PC3 and LNCaP cells, NLRX1 plays a role in ERK signaling, dependent on cell types and context. Serum, EGF, and TGF-β-induced ERK activation are reduced in shNLRX1 PC3 cells, while serum-associated ERK activity remains unchanged in shNLRX1 LNCaP cells. Research on NLRX1's effect on ERK signaling remains limited. NLRX1 disrupts intestinal mucosal function via the ERK/MLC pathway 76, and NLRX1-deficient mice show decreased ERK signaling under hyperoxia 77. PC3 cells show higher basal ERK activation than LNCaP cells 78. While we lack evidence to explain cell-type differences and NLRX1's mechanisms in regulating ERK activity in PCa cells, we speculate that this relates to cell types and cellular context. Future research on NLRX1's mechanism for ERK activation will help clarify its cell-type-specific effects.

In this study, we differentiate AKT and ERK roles in PC3 and LNCaP cells. Studies reveal that NLRX1 participates in PCa cell growth, survival, migration, and invasion, suggesting its positive role in PCa tumorigenesis. Using AKT and ERK inhibitors, our data show AKT's involvement in cell proliferation, migration, invasion, and apoptosis inhibition under serum-free conditions as previously reported 54. ERK signaling mainly contributes to invasion and viability, with less impact on migration, though AKT is less crucial than ERK for cell survival in serum-free conditions. Our findings contrast with studies demonstrating a role for ERK in PCa cell migration under specific conditions 79-81, but are consistent with reports indicating ERK-independent migration 82,83. Moreover, ERK's function in promoting cell growth shows cell-type specificity. Only PC3 cells, but not LNCaP cells, slightly depend on ERK for growth, and this finding is consistent with previous reports 78,84,85. These differential responses may stem from PC3 being androgen receptor-insensitive and LNCaP being receptor-sensitive. This cell-type difference aligns with NLRX1 mediating ERK activation in PC3 but not LNCaP cells. Despite these differences, shNLRX1's ability to block both AKT and ERK explains NLRX1's crucial role in promoting PCa cell functions.

NLRX1 was initially defined as a mitophagy sensor controlling mitochondrial reprogramming and quality 11. While mitophagy-associated gene signature 86 and lncRNA signature 87 offer new approaches for PCa progression, mitophagy's role remains controversial. Studies show mitophagy has a dual role by either attenuating 88 or promoting 89,90 PCa cell death. Consistent with previous findings 91, we observe PCa cell death in serum-free conditions, with no increase in LC3II/I ratio within 24 h. We also note a higher autophagy index in resting PCa cells, suggesting that NLRX1-dependent cell protection under serum-free conditions may be unrelated to mitophagy. However, we cannot exclude mitophagy induction after longer serum-free incubation, as previously shown at 72 h 91.

Serum starvation upregulates NLRX1 transcriptionally and translationally, while complete media reverses this effect, demonstrating the gene's response to serum stress. Although the specific factors responsible for NLRX1 upregulation remain unknown, this response could be mediated by starvation-associated pathways like AMPK, ATF4, and FOXO. Serum starvation reduces mitochondrial ROS and mass, previously reported in PC3 cells during amino acid starvation 92. While control cells showed this response, NLRX1 silencing maintained elevated mitochondrial ROS and mass under serum-starved conditions, suggesting NLRX1 regulates additional stress-response pathways beyond AKT and ERK. Previous research showed that NLRX1 silencing decreased ATP production and impaired cell functions in breast cancer cells 33. Understanding the mechanisms regulating NLRX1 under starvation and its role in PCa cell metabolism related to AKT remains important.

Several studies show NLRX1 mutations in diseases like colitis 93, multiple sclerosis 20, and hepatitis B virus infection 94,95. The missense mutation NLRX1 p.Arg707Cys is associated with rare congenital genetic abnormalities that increase host susceptibility to chronic HBV infection 95. Patients with HBV infection are more prone to carry the NLRX1 mutation p.Arg707Cys than their healthy counterparts. Wild-type NLRX1 obstructed RIG1-MAVS interaction in a competitive manner; however, NLRX1 mutation diminished this inhibitory effect, leading to higher IFN/NF-κB activation and HBV suppression 94. Mutations of 4 critical residues in NLRX1 (ASP677, PHE680, PHE681, and GLU684), which bind with novel identified ligands punicic acid, eleostearic acid, and docosahexaenoic acid, reduce NLRX1's anti-inflammatory action, leading to enhanced colitis in a mouse model 93. Understanding the structure-associated cellular functions of NLRX1 remains an interesting research topic.

This study has some limitations that need to be considered in interpreting the results. Our analysis of NLRX1 expression in PCa relied largely on transcriptomic datasets, which showed variability across platforms, and protein-level validation in well-annotated clinical cohorts was not performed. Thus, the pathological and prognostic significance of NLRX1 requires confirmation by immunohistochemistry in PCa patient samples. Furthermore, mechanistic links between NLRX1 and key PCa pathways, particularly androgen receptor signaling, were not examined, limiting interpretation of its role in disease progression. Finally, most functional findings were derived from *in vitro* systems under serum-deprivation conditions. Together, these limitations highlight important directions for future studies to establish the clinical relevance and mechanistic framework of NLRX1 in PCa.

In conclusion, our findings highlight NLRX1 as a multifaceted tumor-promoting regulator in PCa**.** Silencing of NLRX1 significantly impairs cell proliferation, survival, migration, and invasion, ultimately leading to reduced tumor growth. Mechanistically, these oncogenic roles of NLRX1 are mediated, at least in part, through the activation of AKT and/or ERK signaling pathways. Collectively, we suggest that targeting the NLRX1-AKT axis may represent a promising strategy to modulate the tumor microenvironment and suppress PCa progression, offering potential clinical relevance for future PCa treatment.

## Supplementary Material

Supplementary figures.

## Figures and Tables

**Table 1:** Primer list for real-time PCR

NLRX1-F	CTGCCTCTGCTCTTCAACCT
NLRX1-R	CTCGAAACATCTCCAGCACC
AKT-F	TGGACTACCTGCACTCGGAGAA
AKT-R	GTGCCGCAAAAGGTCTTCATGG
PDK1-F	CACCATGCCAACAGAGGTGTT
PDK1-R	CCTCATTACCCAGCGTGACA
PLK4-F	GTGTATCTGGTATTAGAAATGTG C
PLK4-R	GGAGGTTAGAAAGTGTGAGGTC

**Table 2:** Primer list for constructing NLRX1 plasmids

nlrx1-BamHI-F1	ATGCGGATCCATGAGGTGGGGCCACCATTTGC
nlrx1-BamHI-F2	ATGCGGATCCACTGAAGCTATACAGCGGCAC
nlrx1-XhoI-R2	ATCGCTCGAGTCAGCTTCCAGAGCTTCCCAGC
nlrx1-XhoI-R3	ATCGCTCGAGCTAGATCAGGTTGAAGAGCAGAGG
nlrx1-BamHI-F3	TCTGGGAGGAGGATCCATCAAGGTGGTTCCACGA
nlrx1-NheI-R3	GCCAAGCTGAGCTAGCACA

## Data Availability

All data related to this study are included in this article or will be made available on request.
