# Supplementary material for: NLRX1 Drives Prostate Cancer Progression Through Activation of AKT and ERK Signaling Pathways
Source: Int J Biol Sci. 2026 Apr 16;22(8):4417–40. doi: 10.7150/ijbs.126054 (PMC13138247; doi:10.7150/ijbs.126054)
Supplement: Supplementary file 1 — Supplementary figures. [file ijbsv22p4417s1.pdf]

Supplement

NLRX1 Drives Prostate Cancer Progression Through Activation of AKT and ERK Signaling Pathways

Varsha Rathore[1, 2, 3], Ching-Yuan Cheng[3], Duen-Yi Huang[3], Shao-Peng Chen[3], Liang Huan Wu[3, 4], Jitendra Maharana[1, 5], Chuang-Rung Chang[2], Wan-Wan Lin[3, 6, *]

[1] Chemical Biology and Molecular Biophysics, Taiwan International Graduate Program, Academia Sinica, Taipei 115201, Taiwan

[2] Institute of Biotechnology, College of Life Sciences and Medicine, National Tsing Hua University, Hsinchu 300044, Taiwan.

[3] Department of Pharmacology, College of Medicine, National Taiwan University, Taipei 10051, Taiwan.

[4] Department of Ophthalmology, Cardinal Tien Hospital, New Taipei City 23148, Taiwan.

[5] Institute of Bioinformatics and Structural Biology, College of Life Sciences and Medicine, National Tsing Hua University, Hsinchu 300044, Taiwan.

[6] Graduate Institute of Medical Sciences, Taipei Medical University, Taipei 110, Taiwan.

Submission to: **International Journal of Biological Sciences**

*Corresponding author

Wan-Wan Lin, PhD, Department of Pharmacology, College of Medicine, National Taiwan University, Taipei, Taiwan. Tel: (02)23123456#288315; E-mail: wwllaura1119@ntu.edu.tw

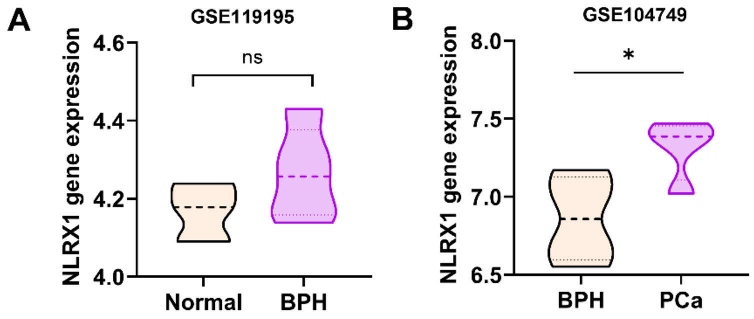

Fig. S1. NLRX1 expression in prostate tissues and clinical subgroups.

(A) NLRX1 mRNA expression in normal prostate tissues and benign prostatic hyperplasia (BPH) samples derived from the GSE119195 GEO dataset. Differences between groups were evaluated using Student's t-test. (B) Comparison of NLRX1 expression between BPH and PCa tissues from a GSE104749 GEO dataset, with statistical significance assessed by Student's t-test.

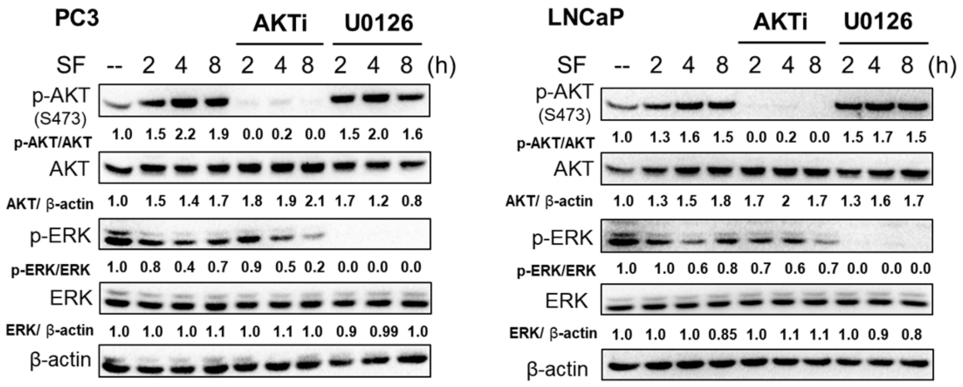

Fig. S2. AKT and ERK signaling dynamics following AKTi and U0126 treatment in PC3 and LNCaP cells.

Cells were treated with AKTi or U0126 under SF conditions. p-AKT, total AKT, p-ERK, and total ERK were analyzed; β-actin served as a loading control. Densitometric analysis of Western blot bands was performed using ImageJ. Protein expression was normalized to β-actin, and values were expressed relative to the 0 h control (set to 1).

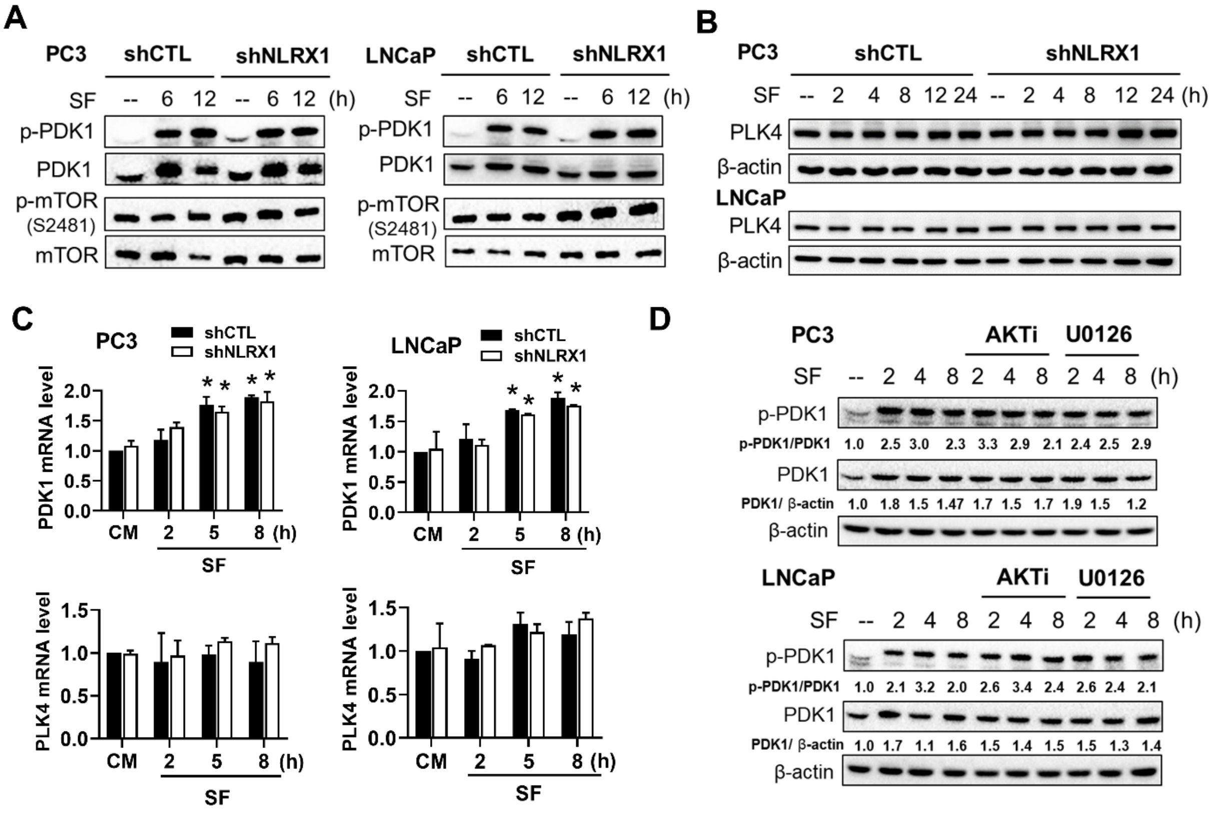

Fig. S3. NLRX1 silencing does not affect PDK1/mTOR/PLK4 signaling under serum-free conditions.

(A, B) PC3 and LNCaP cells were cultured under serum-free (SF) conditions for the indicated times. Immunoblotting was performed to assess p-PDK1, p-mTOR, total mTOR, and PDK1 (A) and PLK4 (B). (C) PDK1 and PLK4 mRNA levels under SF were measured by qRT-PCR. (D) Cells were treated with AKTi or U0126 under SF for the indicated times. p-PDK1, total PDK1, and β-actin were analyzed by immunoblotting. Data are mean ± S.E.M. from independent experiments. *p < 0.05 indicates a significant effect of SF compared to the untreated control group.

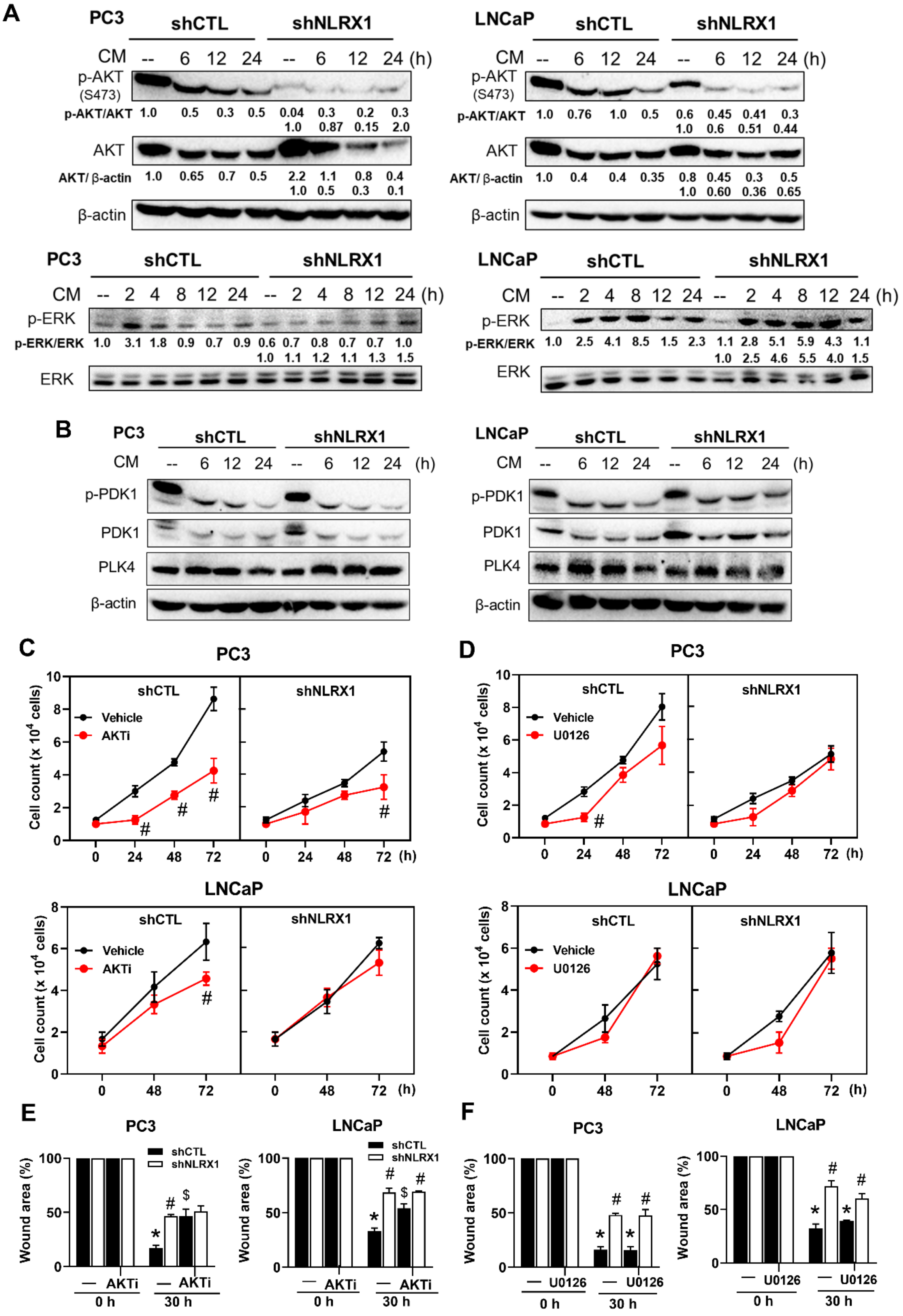
A
PC3
shCTL
shNLRX1
CM
p-AKT (S473)
p-AKT/AKT
AKT
AKT/ β-actin
β-actin
LNCaP
p-ERK
p-ERK/ERK
ERK
B
p-PDK1
PDK1
PLK4
C
D
E
F
Cell count (x 10^4 cells)
Vehicle
AKTi
U0126
Wound area (%)
0 h
30 h
(h)

Fig. S4. NLRX1 silencing inhibits cell growth and migration via AKT suppression.

(A, B) Cells were serum-starved (24 h), then stimulated with CM for the indicated times. p-AKT (S473), total AKT, p-ERK, total ERK, and β-actin were analyzed by immunoblotting. In (A), both quantification of p-AKT/AKT and AKT/□-actin were determined, and values were expressed relative to the 0 h control (set to 1). Moreover, the quantification based on the 0 h response in shNLRX1 cells (set to 1) was also determined. (C, D) Cells were serum-starved overnight, then co-treated with complete medium (CM) and either AKTi (B) or ERKi (U0126) (C). Cell number was determined using trypan blue exclusion. (E, F) Wound healing assays were performed in PC3 and LNCaP cells pre-treated with mitomycin C (1 μg/mL), followed by AKTi (E) or ERKi (F) treatment. Wound closure was monitored microscopically. Data are mean ± S.E.M. from independent experiments. *, $p < 0.05$ indicates a significant effect on the control group (shCTL) compared to the untreated shCTL. #, $p < 0.05$ indicates a significant effect of shNLRX1 compared to shCTL. $, $p < 0.05$ indicates a significant effect of the inhibitor treatment under SF conditions.
